# Optimizing Strategies for Improving Mental Health in Victoria, Australia during the COVID-19 Era: A System Dynamics Modelling Study

**DOI:** 10.3390/ijerph19116470

**Published:** 2022-05-26

**Authors:** Catherine Vacher, Nicholas Ho, Adam Skinner, Jo Robinson, Louise Freebairn, Grace Yeeun Lee, Frank Iorfino, Ante Prodan, Yun Ju C. Song, Jo-An Occhipinti, Ian B. Hickie

**Affiliations:** 1 Brain and Mind Centre, Faculty of Medicine and Health, University of Sydney, Sydney, NSW 2006, Australia; nicholas.ho@sydney.edu.au (N.H.); adam.skinner@sydney.edu.au (A.S.); louise.freebairn@sydney.edu.au (L.F.); grace.lee@sydney.edu.au (G.Y.L.); frank.iorfino@sydney.edu.au (F.I.); a.prodan@westernsydney.edu.au (A.P.); yun.song@sydney.edu.au (Y.J.C.S.); jo-an.occhipinti@sydney.edu.au (J.-A.O.); ian.hickie@sydney.edu.au (I.B.H.); 2 St Vincent’s Clinical School, University of New South Wales, Sydney, NSW 2052, Australia; 3 Orygen, Parkville, VIC 3052, Australia; jo.robinson@orygen.org.au; 4 Centre for Youth Mental Health, University of Melbourne, Melbourne, VIC 3052, Australia; 5 Computer Simulation & Advanced Research Technologies (CSART), Sydney, NSW 2021, Australia; 6 School of Computer, Data and Mathematical Sciences, Western Sydney University, Penrith, NSW 2751, Australia

**Keywords:** mental health, decision analysis, systems modelling

## Abstract

The ongoing COVID-19 pandemic has impacted the mental health of populations and highlighted the limitations of mental health care systems. As the trajectory of the pandemic and the economic recovery are still uncertain, decision tools are needed to help evaluate the best interventions to improve mental health outcomes. We developed a system dynamics model that captures causal relationships among population, demographics, post-secondary education, health services, COVID-19 impact, and mental health outcomes. The study was conducted in the Australian state of Victoria. The model was calibrated using historical data and was stratified by age group and by geographic remoteness. Findings demonstrate that the most effective intervention combination includes economic, social, and health sector initiatives. Assertive post-suicide attempt care is the most impactful health sector intervention, but delaying implementation reduces the potency of its impact. Some evidence-based interventions, such as population-wide community awareness campaigns, are projected to worsen mental health outcomes when implemented on their own. Systems modelling offers a powerful decision-support tool to test alternative strategies for improving mental health outcomes in the Victorian context.

## 1. Introduction

The COVID-19 pandemic has affected many determinants of mental health around the world. While it has been seen as an opportunity to rethink work–life or study–life balance for some, it has also produced a range of negative effects for others, especially the most vulnerable. These adverse effects include a decreased sense of safety, social isolation, uncertainty, unemployment, financial insecurity, grief, fear of illness, increased screen time, and a profound disruption of routines. These factors can generate mental health problems in healthy people and worsen existing mental health disorders in others. A large meta-regression analysis, covering 204 countries from 1 January 2020 to 29 January 2021, estimated that the pandemic caused major depressive disorder and anxiety disorders to increase by 27.6% and 25.6%, respectively [1]. While less susceptible to severe COVID-19, young people have been disproportionately affected by the deleterious psychosocial effects of the pandemic [2]. Social connectedness and social identity are important factors in youth. Unemployment in youth increases rates of depression and anxiety in a durable way that can persist in midlife [3]. A longitudinal study of young people in Ireland showed that young people not in education, employment, or training have an almost threefold increased likelihood of any mental health illness across their lifetime and a sevenfold increased risk of current suicidal ideation [4]. One in five young people seeking help for mental health problems are not in education, employment, or training [5]. As the pandemic continues, strategies are required to help mental health care systems under strain and address key mental health determinants affected by COVID-19 [6]. 

The evolution of the COVID-19 pandemic is uncertain. Since March 2020, SARS-CoV-2 has evolved into five consecutive variants of concern of increasing transmissibility (Alpha originating from the United Kingdom, Beta from South Africa, Gamma from Brazil, Delta from India, and Omicron from multiple countries) [7]. While it is tempting to believe after each wave associated with a new variant that the pandemic is coming to an end, genomic studies show that SARS-CoV-2 evolves at a rate of two mutations per month, interspersed by mutational bursts that create new variants [8]. These mutational bursts could be enabled by infections in immunocompromised patients and by the fact that SARS-CoV-2 can infect a large animal reservoir, including dogs; cats; deer; and, more recently, mice [9,10]. Epidemiologists believe the virus will never be eradicated and will become endemic, but it is unknown whether it will progressively evolve into a more aggressive virus or a benign cold-like virus; the only consensus is that there will be more variants [11].

The study was conducted in the state of Victoria, the hardest hit state of Australia in terms of COVID-19 mortality. Victoria is Australia’s second most populous state and is the most educated state. Victoria has 6.65 million residents (as of 30 June 2021) [12], of which 78.1% reside in major city areas [13]. 70.9% of the population aged 20–64 years holds a post-secondary qualification (as of May 2020) [14]. As of February 2022, Victoria has an unemployment rate of 4.5%, an underemployment rate of 5.9%, and a participation rate of 67.8%, which are slightly better than the national averages (respectively, 4.3%, 6.4%, and 67%). As in most other Australian states, unemployment is higher in urban areas (a 4.8% unemployment rate in major cities areas, which correspond to Greater Melbourne) and for females (4.7% female unemployment rate versus 4.2% for males) [15]. 

Intentional self-harm hospitalisation rates ranged from 77.4 to 97.9 events per 100,000 population between 2012–2013 and 2019–2020 [16], and age-standardised suicide death rates ranged from 10.4 to 11.0 per 100,000 population between 2017 and 2019 [17]. As in almost all other states and territories, these rates were higher outside urban areas: in 2019, the age-standardised suicide death rate was 9.1 per 100,000 population in Greater Melbourne, compared with 16.0 in the rest of Victoria. Using self-harm hospitalisation data at a more granular geographical level (ABS Statistical Area 3) [16] combined with estimated resident population data [18,19], we estimate that in 2019-20, the self-harm hospitalisation rate in Greater Melbourne was 75.9 per 100,000 population compared with 112.0 per 100,000 population in the rest of Victoria.

The Victorian mental health system was extensively evaluated by a Royal Commission established in February 2019 on advice of the Victorian Government. The Royal Commission found that the state’s mental health system was unable to respond to the needs of people suffering from mental illness or psychological distress, unsuitable to meet current and future demands, and in urgent need of reform [20]. The 2019–2020 severe Australian bushfire season and the COVID19 pandemic further heightened the pressures on Victoria’s mental health system and highlighted its limitations. Victorians experienced more than 260 days of lockdowns between March 2020 and October 2021 [21]. Unemployment reached 7.7% in Greater Melbourne in June 2020 and 5.8% in the rest of Victoria in August 2020 [22]. A longitudinal survey reported a statistically significant decrease in social connectedness, subjective well-being, and life satisfaction between May/June 2020 and September 2020 [23]. The last Australian Bureau of Statistics Household Impact of COVID-19 survey, conducted in June 2021 (not a period of lockdown, though some restrictions remained), showed that 26.5% of Victorians experienced high and very high levels of distress, compared with 18% in the rest of Australia [24].

In response to the pandemic, the Australian government implemented temporary economic initiatives to dampen rising unemployment rates (JobKeeper wages subsidy program, 30 March 2020 [25]) and health sector initiatives that allowed patients more frequent access to mental health clinicians and care (Better Access Initiative with Additional COVID-19 MBS mental health support, 9 October 2020 [26]). From September 2020 on, about 20 mental health service centres called ‘Head to Health hubs’ were progressively rolled out in Victoria to provide additional mental health support during the pandemic [27]. The Australian Department of Health also launched a web portal providing access to digital mental health services, including apps, online programs, information resources, and telephone services [28].

The pandemic has highlighted the value of modelling to support the strategic planning of responses to the pandemic as it evolves, such as lockdowns and other mitigation strategies against COVID-19 transmission, vaccination policies, and health care system capacity [29]. Similarly, systems modelling and simulation offer an important tool for planning mental health policies, such as mapping and quantifying the complex mechanisms driving mental health, accounting for the dynamic complexity of feedback processes, and simulating the potentially non-additive effect of intervention combinations beyond traditional analytic approaches [30,31,32,33]. The aim of our study was to model interventions, in order to find the best combinations of health, economic, and social initiatives to improve mental health outcomes post pandemic. The effect of interventions was modelled for the general population and separately for the 15–24 years age group. As rates of self-harm and suicides are higher in regional than urban Australia [34] and the impact of COVID-19 was higher in urban areas (higher infection rates and more stringent public health restrictions), the model was stratified by urban versus regional and remote status.

## 2. Methods

### 2.1. Model Structure

The system dynamics model was developed using Stella Architect version 2.0. It captures the causal relationships between population, demographics, geographic remoteness, post-secondary education, labour force, psychological distress, health services, and suicidal behaviour for the Victorian context and incorporates the impact of the COVID-19 pandemic on these components (Supplementary Figure S1). The model is divided into:

1. A population component that models the changes in population sizes across different age groups and geographic remoteness groups as governed by birth, mortality, overseas migration, and internal migration rates;
2. An education component that captures the commencement, discontinuation, and completion of post-secondary education and vocational studies;
3. An employment component that models the flows between employment, underemployment, unemployment, and not participating in the labour force;
4. A psychopathological vulnerability component that captures the increased risk of developmentally vulnerable children developing mental health disorders as they age into adulthood;
5. A psychological distress component that models the onset of and recovery from moderate to very high psychological distress as measured by the Kessler Psychological Distress Scale (K10) [35] (score 16–50) in the population;
6. A mental health services component that models the flow of psychologically distressed, help-seeking people through various services pathways, including general practitioners, psychiatrists, allied health services, hospitals, and community mental health services;
7. A suicidal behaviour component that models self-harm hospitalisations and suicide deaths; and
8. A COVID-19 pandemic component that models the impact of the pandemic and the resulting public health orders on labour force transitions, overseas migration, psychological distress levels, and mental health services demand and usage.

Components are dynamically coupled so that the parameters and outputs of components inform each other across time. For example, the population component provides population size estimates to calculate the number of people in different labour force states, changes over time in unemployment and underemployment, and rates of psychological distress that then influence service demand and usage rates and the number of suicide attempts and deaths.

### 2.2. Data Sources

We tested the model’s ability to reproduce historical, time-series data extracted from the Australian Bureau of Statistics (population sizes, birth and mortality rates, overseas and internal migration rates [36], employment rates and flows [22,37], education and non-school qualification data [14], psychological distress prevalence [38], family characteristics [39], suicide deaths [17], and the impact of the pandemic on psychological distress prevalence [24]), the Australian Institute of Health and Welfare (intentional self-harm hospitalisations, mental-health-related emergency department presentations (henceforth, mental-health-related ED presentations) [16], and mental health service usage statistics [40]), and the Australian Early Development Census (childhood developmental vulnerability prevalence data [41]). For parameters where no data were available, we relied on Powell’s method [42] to perform constrained optimisation, whereby the optimal values are determined by minimising the mean absolute percent error of the model’s outputs compared to the historical data.

### 2.3. Interventions Tested

We simulated a suite of interventions reflecting economic and health system strengthening policies. Used as a decision-support tool, the model affords the opportunity to explore the impacts of different strategies prior to real-world implementation. The interventions are listed in Table 1 and are described in greater detail in Supplementary Table S1. The tested interventions were selected on the basis of their prominence in national discourse for the mitigation of the impacts of the pandemic on mental health. In addition, they represent a cross-section of strategies covering primary and secondary prevention and targeted versus universal approaches.

The tested interventions are not currently running in Victoria, except for a small scale pilot of post-suicide attempt care [43] and an education support program limited to vocational training [44]. The two initiatives already enacted by the Australian government (the JobKeeper wages subsidy program, active between 30 March 2020 to 28 March 2021, and the Better Access Initiative with Additional COVID-19 MBS mental health sessions, active between 9 October 2020 and 30 June 2022) were implemented in our model as part of the COVID-19 baseline scenario.

### 2.4. Assumptions

Following population projections from Charles-Edwards et al. [45], we implemented an overseas migration reduction effect into the model, resulting in 462,726 fewer Victorians by 2030 compared to a no-pandemic scenario. We assumed that the impact of the COVID-19 on model components began in March 2020, which is when the World Health Organisation declared COVID-19 to be a pandemic [46] and when the significant impacts on the Australian economy began.

### 2.5. Projected Outcomes

The model was used to forecast three adverse mental health outcomes: intentional self-harm hospitalisations, mental-health-related ED presentations, and suicide deaths. We assessed the projected impacts of the pandemic by comparing the cumulative adverse mental health events between March 2020 and March 2026 against a no-pandemic scenario. To assess the projected impact of various interventions, we compared the cumulative number of events in the same period with the intervention enabled against the COVID-19 baseline scenario.

The model was stratified by geographic remoteness and by age. We present results for the Victorian population as a whole, for the Victorian population aged 15–24 years, and for the Victorian population by geographic remoteness (major cities areas of Victoria, which correspond to Greater Melbourne, and regional and remote areas of Victoria).

### 2.6. Sensitivity Analysis

Given that uncertainty in intervention effect estimates may impact model projections, we followed the approach of Atkinson et al. [33] to perform sensitivity analyses on these estimates. Briefly, this approach randomly samples 100 sets of values from a uniform joint distribution of intervention effect parameter values within +/−20% of the default value. The model was run with each of these sets of values and the resulting projections for cumulative adverse mental-health-related outcomes were compared between COVID-19 baseline and intervention-enabled scenarios. All intervals reported in this study were derived from the distributions of model outputs calculated during the sensitivity analysis.

## 3. Results

Table 2 provides the projected change in cumulative adverse mental health events over the period March 2020 to March 2026 as a result of the pandemic. Compared to a scenario where the pandemic never occurred, cumulative intentional self-harm hospitalisations are predicted to increase by 5.81% (2060 more events), mental-health-related ED presentations by 1.81% (6480), and suicide deaths by 4.98% (217). Young adults aged 15–24 years are more impacted than the general population, with increases of 7.69% (829 more events) for self-harm hospitalisations, 2.37% (1786) for mental-health-related ED presentations, and 7.69% (43) for suicide deaths.

Urban areas are projected to experience worse mental health across all three outcomes (intentional self-harm hospitalisations, mental-health-related ED presentations, and suicide deaths) than regional and remote areas (Table 2). Intentional self-harm hospitalisations are projected to increase by 6.14% in major cities areas compared to 5.03% in regional and remote areas, mental-health-related ED presentations by 2.16% compared to 1.04%, and suicide deaths by 5.24% compared to 4.42%. These forecasts take into account the greater impact of the COVID-19 pandemic on population sizes in regional and remotes areas. For major cities areas, the population is projected to grow by 10.77% in a non-pandemic scenario compared to 9.05% for the baseline scenario. In contrast, growth for regional and remote areas is predicted to be 5.09% in a non-pandemic scenario versus 2.65% for the baseline scenario.

**Figure 1:**
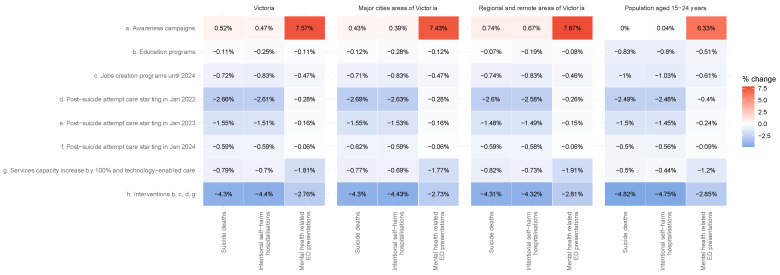
The impact of interventions: a percentage change in the projected cumulative adverse mental health outcomes between March 2020 and March 2026, with several interventions compared to the COVID-19 baseline. The percentage change is calculated from event numbers rounded to the nearest integer.

Figure 1 shows the impact of interventions on mental health outcomes compared with the COVID-19 baseline. All interventions by default start in January 2022, and their impact is measured as a percentage change in cumulative mental health outcomes between March 2020 and March 2026. All 95% intervals, derived from the sensitivity analysis, are displayed in Supplementary Table S3. Regardless of geographic remoteness, post-suicide attempt care is the most effective single intervention to reduce intentional self-harm hospitalisations and suicide deaths. Post-suicide attempt care, consisting of active outreach and contact programs that aim to reduce re-admission, is projected to reduce intentional self-harm hospitalisations by 2.61% (95% interval, (2.14–3.08%)) and suicide deaths by 2.66% (95% interval, (2.18–3.14%)), with similar percentage reductions across urban and regional and remote areas (Figure 1, Supplementary Tables S2 and S3). Delays in implementing post-suicide attempt care reduce the program’s effectiveness (Figure 1), which highlights the importance of offering a rapid response to highly distressed and self-harming individuals.

Surprisingly, one intervention is projected to produce outcomes worse than business-as-usual in Victoria. This intervention consists of public mental health awareness programs aimed at increasing suicide risk recognition, reducing stigma, and increasing help-seeking. Interrogating the model showed that this intervention increases demand on an already at-capacity mental health service system; individuals seeking help and unable to access specialist mental health services become discouraged, which increases psychological distress and the risk of suicidal behaviour.

The most effective intervention for reducing mental-health-related ED presentations is the combination of an increase in mental health services capacities and the implementation of technology-enabled care: doubling the annual rate of increase of mental health consultations (including GP mental health services, psychiatrist and allied services, and community mental health) coupled with technology-enabled care would decrease mental-health-related ED by 1.77% (95% interval, (1.46–2.02%)) in major cities areas and by 1.91% (95% interval, (1.68–2.08%)) in regional and remote areas (Figure 1).

Of note, none of the individual interventions tested in this policy modelling analysis are able to reduce the number of adverse mental health events on-par with a no-pandemic scenario, highlighting that the pandemic necessitates innovative combinations of interventions. Adding economic and social initiatives to the health interventions delivers the best projected results. Financially supporting post-secondary education students to continue their education if they become unemployed as a result of the pandemic would prevent 394 mental-health-related ED presentations (95% interval, (332–456)) in the 15–24 years age group. An employment program designed to double the per capita rate of employment initiation would decrease self-harm hospitalisations by 0.83% (95% interval, (0.61–0.98%)) for the general Victorian population and by 1.03% (95% interval, (0.76–1.23%)) for the 15–24 years age group.

The optimal set of interventions consists of combining these above-described programs: programs aimed at financially supporting post-secondary education students to continue their education, employment programs, an increase in mental health services capacities, technology-enabled care to provide multi-disciplinary and coordinated care, and post-suicide attempt care. This combination is projected to prevent 1650 intentional self-harm hospitalisations (4.40% reduction compared to COVID-19 baseline, 95% interval (3.78–4.92%)), 10,059 mental-health-related ED presentations (2.76%, 95% interval (2.42−3.05%)), and 197 suicide deaths (4.30%, 95% interval (3.7–4.83%)) in Victoria over the period March 2020 to March 2026 (Figure 1, Supplementary Tables S2 and S3).

**Figure 2:**
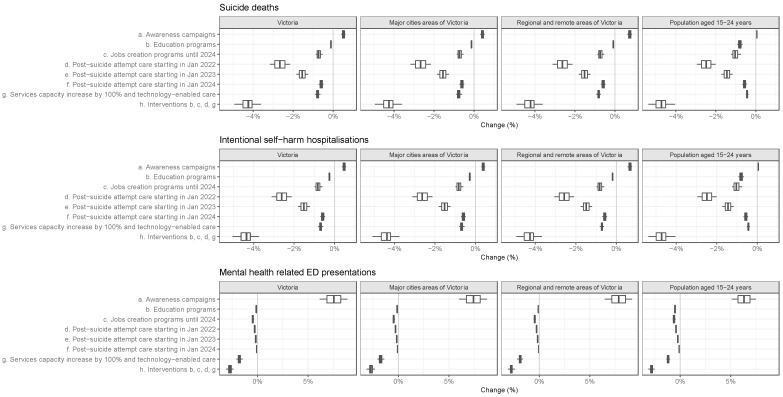
The impact of interventions: a percentage change in projected cumulative adverse mental health outcomes between March 2020 and March 2026, with several interventions compared to the COVID-19 baseline derived from the sensitivity analysis. Note that the horizontal axes are drawn at different scales for the three outcomes to improve legibility.

Sensitivity analysis shows that despite uncertainty surrounding the effectiveness of the real-world implementation of these interventions, most interventions tested here are projected to reduce the number of adverse mental health outcomes and confirms that a combination of multi-sectoral interventions is most effective (Figure 2, Supplementary Table S3).

## 4. Discussion

This study used system dynamics modelling and simulation to test the impact of a range of interventions to improve mental health in the Australian state of Victoria in the context of the COVID-19 pandemic. The model, calibrated using historical data, simulated three main outputs: mental-health-related ED presentations, self-harm hospitalisations, and suicide deaths.

The simulations showed that the combination of an increase in mental health services capacities and the implementation of technology-enabled care can effectively reduce the number of mental-health-related ED presentations. Technology-enabled care involves the use of online technologies to improve the coordination of patient care by multi-disciplinary teams and hence can help increase the efficiency of existing mental health services capacities. In addition, technology-enabled care introduces the key notion of measurement of patient outcomes, where patients’ mental status, progress, or lack of progress can be tracked and acted upon. While technology-enabled care can achieve efficiency gains in mental health services, it provides the best outcomes when it is associated with an increase in mental health services capacities. This highlights the substantial lack of capacity and investment in specialist mental health services [47].

The pandemic has produced different methods of care provision, including telehealth. Many health professionals had long held the view that the therapeutic alliance could only be established face to face, though several studies showed care via telephone or videoconferencing can be as effective [48]. The pandemic can be an opportunity to improve mental health care: telehealth will stay, and technology can be further expanded to provide better continuity of care for patients.

The model projected that self-harm hospitalisations and suicide deaths can be significantly reduced by implementing an active outreach post-attempt care program. While the number of suicides has remained stable in Victoria since the beginning of the pandemic, the rate of ambulance attendances for self-harm rose from 4.1 per 100,000 population in June 2019 to 6.0 in June 2021 [16]. Post-attempt care programs aim to reduce readmission rates with individually tailored counselling and continuity of contact and have been recommended by both the Royal Commission into Victoria’s Mental Health System [20] and the Australian federal government [49]. A limited post-attempt care program (Hospital Outreach Post-suicidal after Engagement (HOPE) program) has been implemented in some parts of Victoria, and the Victorian government has funded a small-scale youth-specific aftercare program [43]. The substantial improvements in self-harm hospitalisations and suicide deaths projected by the model strongly support the extension and scaling of these programs to the whole of Victoria.

Mental health awareness campaigns resulted in the surprising outcome of sharply increasing mental-health-related ED presentations and moderately raising self-harm hospitalisations and suicides. An increase in mental-health-related ED presentations is particularly unadvisable during an ongoing pandemic, where emergency departments may be overwhelmed with surges of COVID-19 patients. Many mental health campaigns have been funded in Victoria and Australia [50,51] as these campaigns look intuitively helpful. However, mental health awareness campaigns are harmful unless they are coupled with investments to cope with the additional demand placed on mental health care services as a consequence of these campaigns. This highlights that the efficacy of mental health awareness campaigns should not only be measured in terms of recall of campaign messages, an improvement in mental health literacy, or the social acceptance of mental disorders [52] but more ecologically in terms of global impact on mental health outcomes such as self-harm behaviour, mental-health-related ED presentations, or other mental health indicators. Our results show the value of system dynamics modelling in pre-empting potential unintended consequences.

The best outcomes were achieved when health interventions were coupled with economic and social interventions, namely, a job-creation program, and an education financial support program targeting post-secondary students (age 15 to 24) who have lost employment. Unemployment in Victoria is currently at a low 4.5% rate, with a participation rate of 67.8% [15]. However, the economy is facing uncertainties. COVID-19 has created supply chain issues; energy prices are increasing; and inflation is at a 40 year-high (7.5%) in the United States and at 5.4% in the United Kingdom and is predicted to rise in other developed countries [53]. The Russia–Ukraine conflict is forecast to decrease GDP growth by 1.08% and increase inflation by 2.47% worldwide over the next 12 months [54]. At the same time, the Australian government, like in many other countries, has stopped its COVID-19 financial support payments [25] and recently ended quantitative easing [55]. The Victorian government has ruled out future lockdowns, but the recent Omicron COVID-19 wave caused a self-imposed lockdown, with a sharp decrease in hours worked [15]. In essence, economic conditions remain uncertain while the safety nets are removed. Unemployment is associated with greater psychological distress and has a deleterious effect on mental health [56,57]. The impact of unemployment on mentally vulnerable individuals leads not only to the loss of financial security but also the loss of personal work relationships, daily routine structures, a sense of purpose, and a sense of self. A period of unemployment can have long lasting effects on mental health, and our modelling analysis projects a substantial positive effect on mental health with an employment creation program of only two years duration.

An intervention providing financial assistance to students (15–24 years age group) to help them stay in post-secondary education, even if they lose their employment, was projected to lower the rate of self-harm hospitalisations. Young people with very high psychological distress or early onset of mental health problems are at higher risk of not making the transition from school to employment [58]. Higher educational attainment is associated with better mental health [59]. The pandemic has particularly affected youth, and education provides at a minimum better future financial resources and more social support. The impact of education on population mental health is likely underestimated here due to the short simulation time horizon and the focus of the program limited to financially supporting students to remain in study. The modelled program did not include expansions to the quantity and quality of education and training. This will be further explored elsewhere.

With the exception of the pilot of the HOPE post-attempt care program, we are not aware of prospective research studies in Victoria that implemented the simulated interventions and measured their impact on mental health outcomes. The pilot of the HOPE post-attempt care program measured improvements in outcome and session rating scales after intervention but did not measure the impacts on the rates of re-presentation to services for self-harm [43]. This highlights the role of modelling to help understand likely impacts on mental health outcomes and inform mental health system strengthening.

Combining the health initiatives (post-attempt care, an increase in services capacity and technology-enabled care) and socio-economic interventions (education and job creation programs) produces the best benefits in terms of population mental health. Increasing the services capacity of GPs, psychiatrists, psychologists, mental health nurses, and other allied health professionals, while important, should also consider how best to facilitate multidisciplinary care. Models being trialed in the Australian context include Head to Health hubs and youth mental health hubs such as headspace [27,60]. Technology-enabled care can further increase services capacity by improving the quality and efficiency of services through delivery of a measurement-based model of care and coordination of care provided by these multidisciplinary teams. Altogether, the coupling of services capacity increase with technology-enabled care has the potential to greatly improve the quality and availability of mental health care and is projected to significantly decrease the number of mental-health related ED presentations. To markedly reduce self-harm hospitalisations and suicides, a targeted approach is preferable; the model supports the state-wide extension of the post-attempt care initiatives [43] that have been piloted in Victoria. Crucially, the socio-economic interventions of student financial support and job creation further improve mental health outcomes. The employment-support program enacted by the Australian government during the start of the pandemic has ended, and the only education financial support measure currently active is a program limited to vocational training [44]. The proposed education financial support and job creation interventions complement the health interventions and act to both prevent the onset of common mental health disorders and better support individuals with pre-existing disorders to avoid deterioration.

## 5. Limitations

This study has a number of limitations. First, the model draws input from population health surveys, demographic and economic data for small geographical areas, and Medicare Benefits schedule data that might vary in quality. Parameter estimation via constrained optimisation and local verification were used to identify plausible estimates when these data were not available from published research or other sources. These are made transparent in the supplementary materials, and the model can be updated as new evidence and data become available. Second, suicides are potentially underestimated due to misclassification of suicides as unintentional injuries or events of undetermined intents. Third, self-harm hospitalisations, which only represent self-harm attempts requiring medical interventions, might underestimate the true number of self-harm attempts in Victoria.

## 6. Conclusions

As the world is facing uncertainty in terms of pandemic trajectory and economic outlook, it is important to invest in strengthening mental health care and social systems. This allows for supporting individuals already struggling with mental disorders, as well as building a resilient population. As many countries belonging to the Organisation for Economic Co-operation and Development (OECD) have accumulated a large government debt to provide financial assistance during the pandemic, resources are limited, and interventions need to be carefully planned for optimal outcomes. Systems modelling and simulation, which have been largely used for strategic planning to mitigate the impact of the pandemic, constitute an important decision tool for testing scenarios to achieve the optimal mental health outcomes. Our modelling, applied to the state of Victoria, forecasts the best results with a combination of health, social, and economic interventions.

## Figures and Tables

**Table 1:** Interventions examined in the simulations (further details are provided in Supplementary Table S1). All interventions by default start in January 2022.

Intervention	Description
a. Awareness programs	Population-wide mental health awareness programs aimed at reducing stigma, improving the recognition of suicide risk, and encouraging help-seeking. Default duration: 5 years.
b. Education programs	Programs providing financial support to post-secondary students (age 15 to 24) who have become unemployed due to the COVID-19 pandemic, enabling them to continue studying. Default duration: 5 years.
c. Job creation program	Programs designed to increase the per capita rate of employment initiation. Default duration: 2 years.
d. Post-suicide attempt care	An active outreach and contact program that aims to reduce re-admission in those presenting to services after a suicide attempt. It includes individually tailored contact, solution focused counselling, and actions to ensure adherence to follow-up treatments and continuity of contact. Default duration: till end of simulation.
e. Services capacity growth	GP mental health services, psychiatrist and allied services, and community mental health: multiplies (by 2 by default) the annual rate of increase in the total number of consultations that can be completed per week. Default duration: till end of simulation.
f. Technology-enabled, measurement-based care	Online technology to facilitate the delivery of a multidisciplinary team-based care, where medical and allied health professionals consider all treatment options, collaboratively develop an individual care plan for each patient, and measure outcomes. Default duration: till end of simulation.

**Table 2:** Projected cumulative adverse mental health events between March 2020 and March 2026, comparing a no-pandemic scenario against baseline with COVID-19. Event numbers are rounded to the nearest integer.

	No COVID-19	COVID-19 Baseline	Change	% Change
**Victoria (all ages)**				
Suicide deaths	4361	4578	217	4.98%
Intentional self-harm hospitalisations	35,468	37,528	2060	5.81%
Mental-health-related ED presentations	358,580	365,060	6480	1.81%
**Population aged 15–24 years**				
Suicide deaths	559	602	43	7.69%
Intentional self-harm hospitalisations	10,782	11,611	829	7.69%
Mental-health-related ED presentations	75,226	77,012	1786	2.37%
**Major cities areas of Victoria**				
Suicide deaths	3070	3231	161	5.24%
Intentional self-harm hospitalisations	24,878	26,406	1528	6.14%
Mental-health-related ED presentations	246,643	251,965	5322	2.16%
**Regional and remote areas of Victoria**				
Suicide deaths	1290	1347	57	4.42%
Intentional self-harm hospitalisations	10,590	11,123	533	5.03%
Mental-health-related ED presentations	111,937	113,096	1159	1.04%

## Data Availability

The data supporting the findings of this study are available within the article and its supplementary materials. Details of data sources used for model parameterisation and calibration are provided in the Supplementary Materials section.

## Supplementary Materials

The following supporting information can be downloaded at: https://www.mdpi.com/article/10.3390/ijerph19116470/s1, Figure S1: an overview of the causal structure of the system dynamics model.; Figure S2: adverse mental health outcome estimates derived from the model and the corresponding historical data from the Australian Bureau of Statistics (ABS) and the Australian Institute of Health and Welfare (AIHW); Table S1: interventions and default parameter values. Parameter values can be modified via an interactive dashboard to assess the impact of different parameter values on simulated outputs.; Table S2: the differences in projected cumulative adverse mental health events between March 2020 and March 2026 in Victoria, major cities areas of Victoria, regional and remote areas of Victoria, and Victorians aged 15–24 years. Outcomes are compared to COVID-19 baseline after various interventions; Table S3: the summary statistics for the projected reductions in cumulative adverse mental health events between March 2020 and March 2026 from the sensitivity analysis; Table S4: the numerical inputs and data sources. Inputs highlighted in blue were varied in the sensitivity analysis. References [61,62,63,64,65,66] are cited in the supplementary materials.
