# Supplementary material for: Optimizing Strategies for Improving Mental Health in Victoria, Australia during the COVID-19 Era: A System Dynamics Modelling Study
Source: Int J Environ Res Public Health. 2022 May 26;19(11):6470. doi: 10.3390/ijerph19116470 (PMC9180267; doi:10.3390/ijerph19116470)
Supplement: Supplementary file 1 [file ijerph-19-06470-s001.zip › ijerph-1693355-supplementary.pdf]

SUPPLEMENTARY MATERIAL

Supplementary Figure S1. Overview of the causal structure of the system dynamics model.

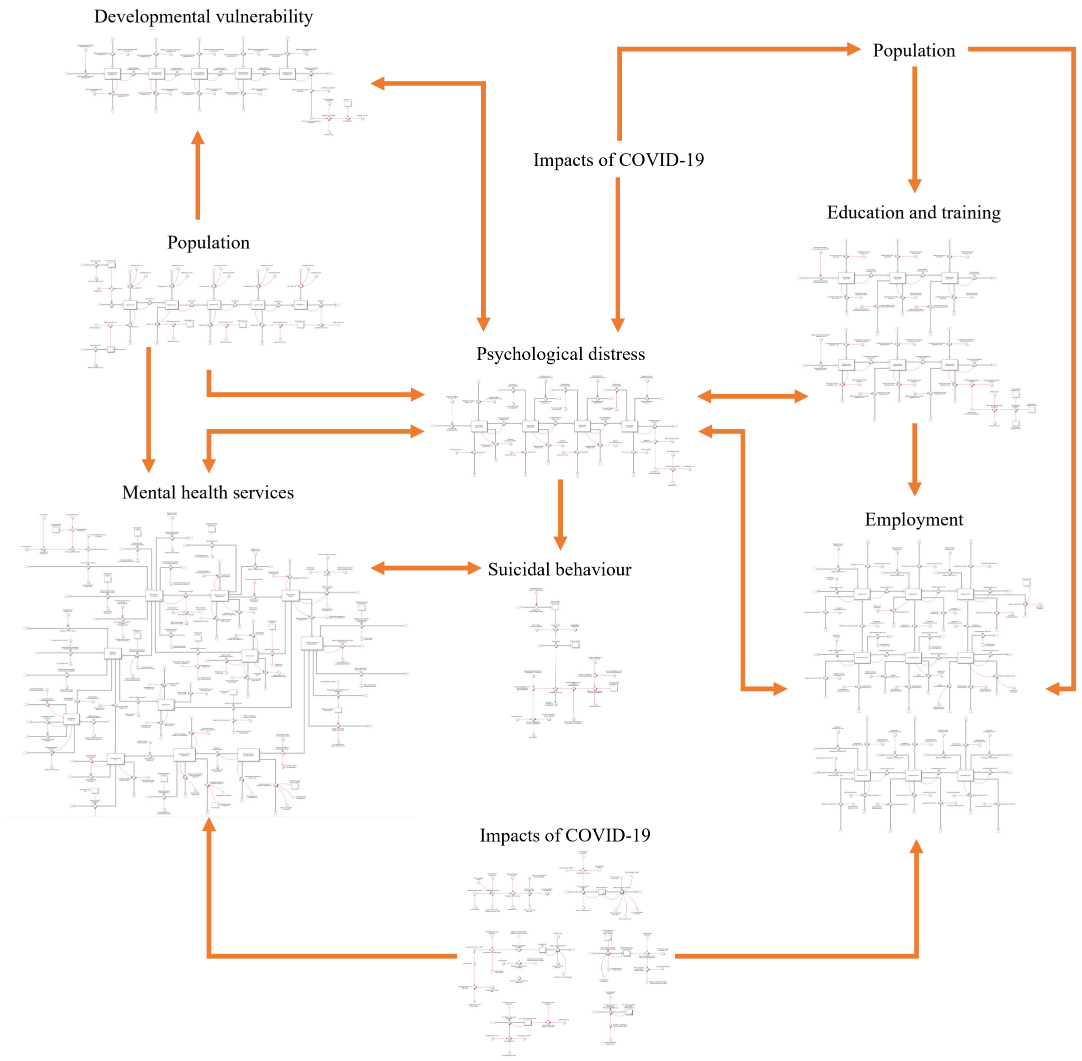

Supplementary Figure S2. Adverse mental health outcome estimates derived from the model and the corresponding historical data from the Australian Bureau of Statistics (ABS) and the Australian Institute of Health and Welfare (AIHW).

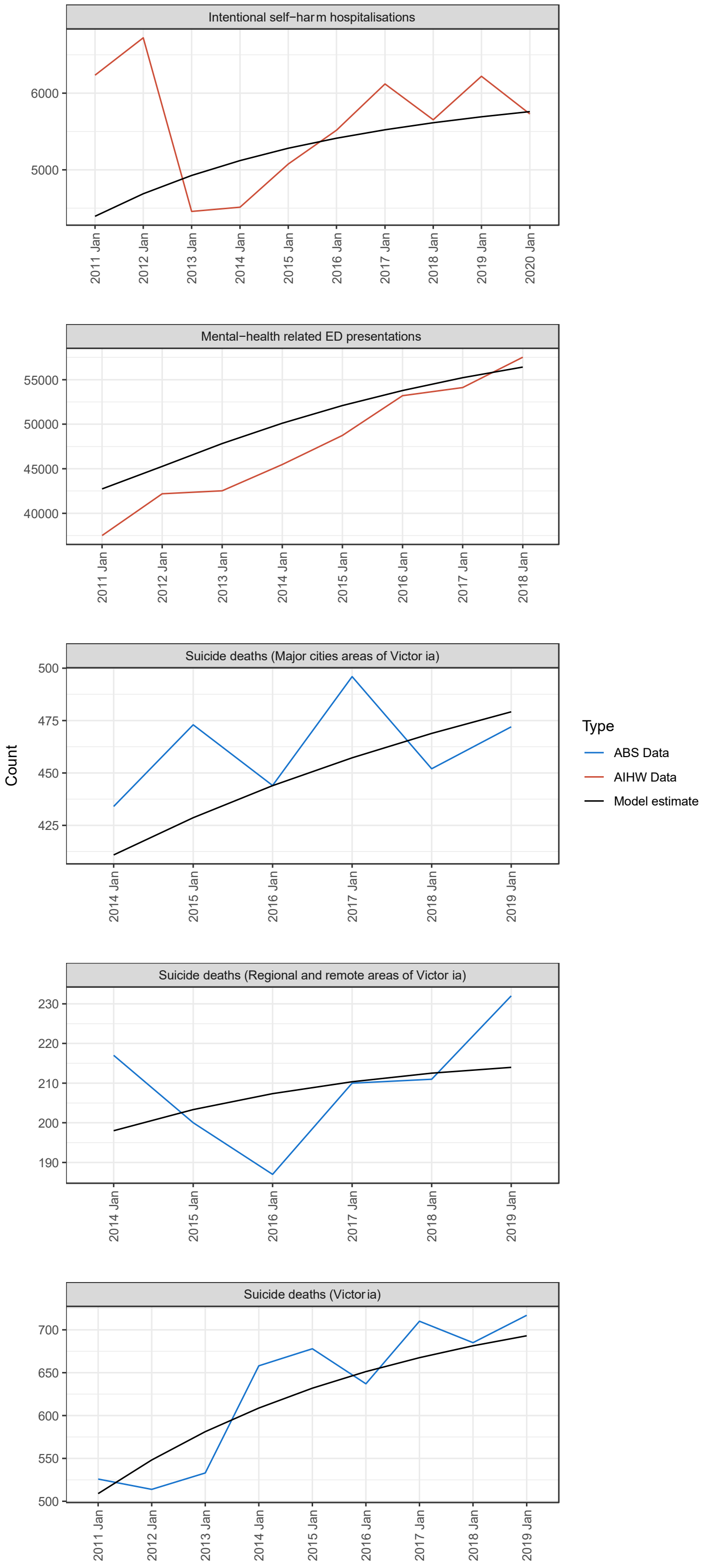

Supplementary Table S1. Interventions and default parameter values. Parameter values can be modified via an interactive dashboard to assess the impact of different parameter values on simulated outputs.

Intervention	Description
**a. Awareness programs**	Population-wide mental health education programs aimed at reducing stigma, improving recognition of suicide risk, and encouraging help-seeking. This intervention increases the per capita rates at which people perceive a need for mental health services and seek help from a general practitioner or online services. Parameters that can be modified are:<br><br>*Starting year* — the year in which mental health awareness campaigns commence (the default is 2022, or January 2022).<br><br>*Implementation time (years)* — the time after commencement required for mental health awareness campaigns to be fully implemented (the default is 0.167 years, or 2 months).<br><br>*Program duration (years)* — the duration of mental health awareness campaigns (the default is 5 years).<br><br>*Effect on engagement* — the multiplicative effect of mental health awareness campaigns on the per capita rates that people perceive a need for mental health care, seek help from a general practitioner, or access online services. The default value (1.585) is derived from Jorm et al. (2003) .<br><br>*Effect decay rate per year* — the fractional rate per year at which the effect on engagement decreases to a value of 1 (i.e., no effect) after mental health awareness campaigns end. The default value (1) implies that the effect on engagement would decrease to a value of 1 in 1 year given the initial rate of decline (i.e., the rate immediately after awareness campaigns end). Note that the rate of decline in the effect of awareness campaigns itself declines as the effect approaches a value of 1, so the actual time required for the effect to decay completely will generally be greater than the inverse of the decay rate specified.
**b. Education programs**	Programs providing financial support to students who have become unemployed due to the COVID-19 pandemic, enabling them to continue studying. This intervention reduces the per capita rate that students discontinue post-secondary study as a direct result of job loss. The per capita rate of enrolment in post-secondary study among people aged 15-24 years can also be modified. Parameters that can be modified are:<br><br>*Starting year* — the year in which education programs commence (the default is 2022, or January 2022).<br><br>*Implementation time (years)* — the time required for education programs to be fully implemented (the default is 0.167 years, or 2 months).

	Program duration (years) — the duration of education programs (the default is 5 years).<br><br>*Effect on discontinuation* — the multiplicative effect of education programs on the proportion of students discontinuing post-secondary study due to job loss. The default value (0.1) assumes that education programs reduce the proportion of students discontinuing study after becoming unemployed by 90%.<br><br>*Effect on enrolment* — the multiplicative effect of education programs on the per capita enrolment rate for 15-24-year-olds. For this intervention, we used a value of 2 to increase the per capita enrolment rate for 15-24-year-olds by 100%.
**c. Jobs creation program**	Programs designed to increase the per capita rate of employment initiation. Parameters that can be modified are:<br><br>*Starting year* — the year in which jobs creation programs commence (the default is 2022, or January 2022).<br><br>*Implementation time (years)* — the time required for jobs creation programs to be fully implemented (the default is 0.167 years, or 2 months).<br><br>*Program duration (years)* — the duration of jobs creation programs (the default is 2 years which will end the Jobs creation program in January 2024).<br><br>*Effect on employment initiation* — the multiplicative effect of employment programs on the per capita employment initiation rate. For this intervention, we used a value of 2 to increase the rate at which people secure employment by 100%.
**d. Post-suicide attempt care**	Post-attempt care is an active outreach and enhanced contact program that aims to reduce re-admission in those presenting to services after a suicide attempt. It includes individually tailored contact, solution focused counselling, and motivations to ensure adherence to follow-up treatments and continuity of contact. Parameters that can be modified are:<br><br>*Starting year* — the year in which post-attempt care programs commence (the default is 2022, or January 2022).<br><br>*Implementation time (years)* — the time after commencement required for post-attempt care programs to be fully implemented (the default is 2 years).<br><br>*Program duration (years)* — the duration of post-attempt care programs (the default is set to 1000 years, ensuring that programs remain in place until the end of the simulation).<br><br>*Maximum rate* — the maximum proportion of patients hospitalised for a suicide attempt receiving post-attempt care. The default value (0.7) assumes that post-attempt care will be provided to 70% of patients hospitalised for a suicide attempt when post-attempt care programs are fully implemented.<br><br>*Post-attempt care effect* – the proportion of potential repeat suicide attempts expected among patients receiving post-attempt

	care. The default value (0.398) implies that 39.8% of repeat attempts that would have occurred without post-attempt care actually occur when post-attempt care is provided; i.e., post-attempt care is assumed to prevent 60.2% of potential repeat suicide attempts. The default estimate is derived from Hvid et al. (2011)<br><br>*Repeat self-harm rate per year* – the probability that a person will self-harm in the year after a suicide attempt without post-attempt care. The default value (0.179) implies that 17.9% of people hospitalised for self-harm will re-attempt within 1 year (i.e., assuming they don't receive post-attempt care); this estimate is derived from Carroll, Metcalfe, and Gunnell (2014)
**e. Services capacity growth**	*GP mental health services* — multiplies the annual rate of increase in the total number of mental health-related GP consultations that can be completed per week. The default value (1) corresponds to the business as usual case, in which services capacity continues to increase at the current rate, estimated using Medicare Benefits Schedule (MBS) data for 2011-2019 assuming services were operating at (near-) maximum capacity over this period. For this intervention, we increased the annual rate of increase by 100% (i.e. set the value to 2).<br><br>*Psychiatrist and allied services* — multiplies the annual rate of increase in the total number of psychiatrist and allied services that can be provided per week. The default value (1) corresponds to the business as usual case, in which services capacity continues to increase at the current rate, estimated using Medicare Benefits Schedule (MBS) data for 2011-2019 assuming services were operating at (near-) maximum capacity over this period. For this intervention, we increased the annual rate of increase by 100% (i.e. set the value to 2).<br><br>*Community mental health* — multiplies the annual increase in the total number of community mental health service contacts that can be provided per week. The default value (1) corresponds to the business as usual case, in which services capacity continues to increase at the current rate, estimated using data for the period 2011-2019 published by the Australian Institute of Health and Welfare (available at: https://www.aihw.gov.au/reports-data/health-welfare-services/mental-health-services/data). For this intervention, we increased the annual rate of increase by 100% (i.e. set the value to 2).<br><br>This multiplicative increase in services capacity growth rates commences in January 2022 and this remains in place until the end of the simulation.
**f. Technology-enabled, measurement-based care**	Technology-enabled, measurement-based care involves the use of online technology to facilitate delivery of multidisciplinary team-based care, in which medical and allied health professionals consider all relevant treatment options and collaboratively develop an individual treatment and care plan for each patient. Online technology improves coordination of care and facilitates communication between medical and allied health professionals, as each health professional involved in the care of a patient has access to the same information about that patient's treatment history.<br><br>Parameters that can be modified in this intervention are:<br><br>*Starting year* — the year in which technology-enabled, measurement-based care is introduced (the default is 2022, or January 2022).

	Implementation time (years) — the time required for technology-enabled, measurement-based care to be fully implemented (the default is 2 years).<br><br>*Program duration (years)* — the duration of investment in technology-enabled, measurement-based care (the default is set to 1000 years, ensuring that investment continues until the end of the simulation).<br><br>*Maximum rate per service* — the maximum proportion of mental health services provided that involve technology-enabled, measurement-based care. This proportion will depend on the number of medical and allied health professionals adopting online care coordination technologies, as well as the number of patients consenting to the use of these technologies in the management of their care (i.e., take-up among service providers and patients). The default value (0.7) assumes that technology-enabled, measurement-based care will be provided in 70% of mental health services completed when fully implemented.<br><br>*Effect on recovery rate* — the multiplicative effect of technology-enabled coordinated care on the per-service recovery rate (i.e., the probability that a patient's level of psychological distress will decrease after receiving treatment). The default estimate (1.177) is derived from Woltmann et al. (2012), and implies that technology-enabled coordinated care increases the per-service probability of a reduction in psychological distress by 17.7%.<br><br>*Effect on referral rate* — the multiplicative effect of technology-enabled, measurement-based care on general practitioners' rates of referral to specialised mental health services (psychiatrists and allied mental health services). The default value (1.266) implies that technology-enabled, measurement-based care increases the per-consultation probability that a general practitioner will refer a patient with moderate to very high psychological distress to specialised psychiatric care by 26.6%, and is derived from Badamgarav et al. (2003).<br><br>*Effect on disengagement* — the multiplicative effect of technology-enabled, measurement-based care on per capita rates of disengagement from mental health services (including disengagement while waiting for services and disengagement resulting from dissatisfaction with services received). The default estimate (0.520) is derived from Badamgarav et al. (2003), and implies that technology-enabled, measurement-based care reduces rates of disengagement by 48.0%.
**Employment programs**	As employment programs were implemented by the Australian government in response to the COVID-19 pandemic, this intervention was enabled for the COVID-19 baseline scenario model calibration.<br><br>These employment programs are designed to stem rapidly increasing unemployment due to the COVID-19 pandemic (e.g., the JobKeeper Payment). This intervention reduces the increase in the per capita job loss rate resulting directly from the pandemic. The per capita rate of employment initiation can also be increased (or decreased); however, the default settings assume that employment programs have no direct effect on employment initiation. Parameters that can be modified are:<br><br>*Starting year* — the year in which employment programs commence (the default is 2020.33, or May 2020).

	Implementation time (years) — the time required for employment programs to be fully implemented (the default is 0.167 years, or 2 months).<br><br>*Program duration (years)* — the duration of employment programs (the default is 0.917 years, 11 months. With the starting year set at 2020.33, this will set Employment programs to end in April 2021).<br><br>*Effect on job loss* — the multiplicative effect of employment programs on the increase in the job loss rate due to the COVID-19 pandemic. The default value (0.56) assumes that employment programs will reduce the increase in the per capita job loss rate by 44% (Australian Bureau of Statistics, 2020).<br><br>*Effect on employment initiation* — the multiplicative effect of employment programs on the per capita employment initiation rate. The default value (1) assumes no effect of employment programs on employment initiation.
**Better Access**	As this program was implemented by the Australian government in response to the COVID-19 pandemic, this intervention was enabled for the COVID-19 baseline scenario model calibration.<br><br>Reform of the existing *Better Access to Psychiatrists, Psychologists and General Practitioners through the MBS* (Better Access) initiative to provide patients with access to a greater number of specialised mental health care consultations per year. This intervention increases the flow of people with a perceived need for mental health care into psychiatrist and allied mental health services. Parameters that can be modified are:<br><br>*Starting year* — the year in which the reformed Better Access initiative commences (the default is 2020.75, or October 2020).<br><br>*Implementation time (years)* — the time after commencement required for the reformed Better Access initiative to be fully implemented (the default is 0.167 years, or 2 months).<br><br>*Program duration (years)* — the duration of the reformed Better Access initiative. The default is set to 1.75 years which sets the Better Access program to end in June 2022).<br><br>*Services per week* — the average number of specialised mental health care services provided per patient per week. The default value (1) assumes that patients attend 1 consultation per week, so that a patient attending a total of 4 consultations (for example) is assumed to do so over a period of 4 weeks.<br><br>*Additional services per patient* — the mean number of additional specialised mental health care services provided per patient per year under the reformed Better Access scheme. The default value (4) assumes that patients will attend an additional 4 consultations per year when the cap on the number of consultations per patient is increased.

Supplementary Table S2. Differences in projected cumulative adverse mental health events between March 2020 and March 2026 in Victoria, major cities areas of Victoria, regional and remote areas of Victoria, and Victorians aged 15-24 years. Outcomes are compared to COVID-19 baseline after various interventions. Events are rounded to the nearest integer and percent reduction is calculated from these rounded events numbers.

	Suicide deaths			Intentional self-harm hospitalisations			Mental-health related ED presentations		
	**Count**	**Prevented**	**% Reduction**	**Count**	**Prevented**	**% Reduction**	**Count**	**Prevented**	**% Reduction**
**Victoria**									
Baseline	4,578			37,528			365,060		
a. Awareness campaigns	4,602	-24	-0.52%	37,706	-178	-0.47%	392,686	-27626	-7.57%
b. Education programs	4,573	5	0.11%	37,433	95	0.25%	364,665	395	0.11%
c. Jobs creation programs until 2024	4,545	33	0.72%	37,218	310	0.83%	363,357	1703	0.47%
d. Post-suicide attempt care starting in Jan 2022	4,456	122	2.66%	36,548	980	2.61%	364,054	1006	0.28%
e. Post-suicide attempt care starting in Jan 2023	4,507	71	1.55%	36,960	568	1.51%	364,475	585	0.16%
f. Post-suicide attempt care starting in Jan 2024	4,551	27	0.59%	37,308	220	0.59%	364,832	228	0.06%
g. Services capacity increase by 100% and technology-enabled care	4,542	36	0.79%	37,267	261	0.70%	358,448	6612	1.81%
h. Interventions b, c, d, g	4,381	197	4.30%	35,878	1650	4.40%	355,001	10059	2.76%
**Major cities areas of Victoria**									
Baseline	3,231			26,406			251,965		
a. Awareness campaigns	3,245	-14	-0.43%	26,508	-102	-0.39%	270,686	-18721	-7.43%
b. Education programs	3,227	4	0.12%	26,331	75	0.28%	251,658	307	0.12%
c. Jobs creation programs until 2024	3,208	23	0.71%	26,187	219	0.83%	250,777	1188	0.47%
d. Post-suicide attempt care starting in Jan 2022	3,144	87	2.69%	25,712	694	2.63%	251,257	708	0.28%
e. Post-suicide attempt care starting in Jan 2023	3,181	50	1.55%	26,003	403	1.53%	251,552	413	0.16%
f. Post-suicide attempt care starting in Jan 2024	3,211	20	0.62%	26,249	157	0.59%	251,803	162	0.06%
g. Services capacity increase by 100% and technology-enabled care	3,206	25	0.77%	26,225	181	0.69%	247,508	4457	1.77%
h. Interventions b, c, d, g	3,092	139	4.30%	25,236	1170	4.43%	245,080	6885	2.73%
**Regional and remote areas of Victoria**									
Baseline	1,347			11,123			113,096		

a. Awareness campaigns	1,357	-10	-0.74%	11,197	-74	-0.67%	122,000	-8904	-7.87%
b. Education programs	1,346	1	0.07%	11,102	21	0.19%	113,007	89	0.08%
c. Jobs creation programs until 2024	1,337	10	0.74%	11,031	92	0.83%	112,579	517	0.46%
d. Post-suicide attempt care starting in Jan 2022	1,312	35	2.60%	10,836	287	2.58%	112,797	299	0.26%
e. Post-suicide attempt care starting in Jan 2023	1,327	20	1.48%	10,957	166	1.49%	112,923	173	0.15%
f. Post-suicide attempt care starting in Jan 2024	1,339	8	0.59%	11,059	64	0.58%	113,029	67	0.06%
g. Services capacity increase by 100% and technology-enabled care	1,336	11	0.82%	11,042	81	0.73%	110,940	2156	1.91%
h. Interventions b, c, d, g	1,289	58	4.31%	10,642	481	4.32%	109,921	3175	2.81%
**Population aged 15-24 years**									
Baseline	602			11,611			77,012		
a. Awareness campaigns	602	0	0.00%	11,616	-5	-0.04%	81,884	-4872	-6.33%
b. Education programs	597	5	0.83%	11,518	93	0.80%	76,618	394	0.51%
c. Jobs creation programs until 2024	596	6	1.00%	11,491	120	1.03%	76,540	472	0.61%
d. Post-suicide attempt care starting in Jan 2022	587	15	2.49%	11,323	288	2.48%	76,701	311	0.40%
e. Post-suicide attempt care starting in Jan 2023	593	9	1.50%	11,443	168	1.45%	76,831	181	0.24%
f. Post-suicide attempt care starting in Jan 2024	599	3	0.50%	11,546	65	0.56%	76,941	71	0.09%
g. Services capacity increase by 100% and technology-enabled care	599	3	0.50%	11,560	51	0.44%	76,088	924	1.20%
h. Interventions b, c, d and e combined	573	29	4.82%	11,060	551	4.75%	74,816	2196	2.85%

Supplementary Table S3. Summary statistics for the projected reductions in cumulative adverse mental health events between March 2020 and March 2026 from the sensitivity analysis.

	Suicide deaths			Intentional self-harm hospitalisations			Mental-health related ED presentations		
	**Min**	**Max**	**Median (95% Interval)**	**Min**	**Max**	**Median (95% Interval)**	**Min**	**Max**	**Median (95% Interval)**
**Victoria**									
a. Awareness campaigns	-0.62	-0.4	-0.52 (-0.62, -0.41)	-0.57	-0.37	-0.47 (-0.56, -0.37)	-8.89	-6.16	-7.57 (-8.83, -6.23)
b. Education programs	0.09	0.13	0.11 (0.09, 0.13)	0.21	0.3	0.26 (0.21, 0.29)	0.09	0.13	0.11 (0.09, 0.12)
c. Jobs creation programs until 2024	0.53	0.86	0.73 (0.54, 0.86)	0.6	0.99	0.83 (0.61, 0.98)	0.34	0.56	0.47 (0.35, 0.55)
d. Post-suicide attempt care starting in Jan 2022	2.15	3.16	2.66 (2.18, 3.14)	2.11	3.1	2.61 (2.14, 3.08)	0.22	0.33	0.28 (0.23, 0.32)
e. Post-suicide attempt care starting in Jan 2023	1.25	1.84	1.54 (1.26, 1.82)	1.22	1.8	1.51 (1.24, 1.79)	0.13	0.19	0.16 (0.13, 0.19)
f. Post-suicide attempt care starting in Jan 2024	0.48	0.72	0.6 (0.49, 0.71)	0.47	0.7	0.59 (0.48, 0.7)	0.05	0.07	0.06 (0.05, 0.07)
g. Services capacity increase by 100% and technolo gy-enabled care	0.63	0.91	0.78 (0.66, 0.88)	0.57	0.8	0.69 (0.59, 0.78)	1.47	2.1	1.81 (1.55, 2.02)
h. Interventions b, c, d, g	3.61	4.94	4.24 (3.7, 4.83)	3.74	5.04	4.35 (3.78, 4.92)	2.37	3.11	2.73 (2.42, 3.05)
**Major cities areas of Victoria**									
a. Awareness campaigns	-0.53	-0.31	-0.42 (-0.52, -0.32)	-0.48	-0.29	-0.39 (-0.48, -0.29)	-8.77	-6.01	-7.43 (-8.7, -6.09)
b. Education programs	0.1	0.14	0.12 (0.1, 0.14)	0.24	0.33	0.28 (0.24, 0.33)	0.1	0.14	0.12 (0.1, 0.14)
c. Jobs creation programs until 2024	0.53	0.86	0.72 (0.54, 0.85)	0.6	0.99	0.83 (0.61, 0.98)	0.34	0.56	0.47 (0.35, 0.56)
d. Post-suicide attempt care starting in Jan 2022	2.17	3.18	2.68 (2.19, 3.15)	2.13	3.12	2.63 (2.15, 3.09)	0.23	0.33	0.28 (0.23, 0.33)
e. Post-suicide attempt care starting in Jan 2023	1.26	1.85	1.56 (1.27, 1.84)	1.23	1.82	1.53 (1.25, 1.8)	0.13	0.19	0.16 (0.13, 0.19)
f. Post-suicide attempt care starting in Jan 2024	0.49	0.72	0.61 (0.49, 0.72)	0.48	0.71	0.59 (0.48, 0.7)	0.05	0.08	0.06 (0.05, 0.08)
g. Services capacity increase by 100% and technolo gy-enabled care	0.6	0.9	0.76 (0.63, 0.88)	0.54	0.8	0.68 (0.56, 0.78)	1.4	2.06	1.75 (1.46, 2.02)
h. Interventions b, c, d, g	3.61	4.95	4.25 (3.69, 4.85)	3.76	5.08	4.37 (3.79, 4.98)	2.32	3.14	2.72 (2.36, 3.04)
**Regional and remote areas of Victoria**									
a. Awareness campaigns	-0.85	-0.62	-0.74 (-0.85, -0.63)	-0.77	-0.56	-0.67 (-0.77, -0.57)	-9.18	-6.48	-7.87 (-9.11, -6.56)
b. Education programs	0.07	0.1	0.08 (0.07, 0.1)	0.16	0.22	0.19 (0.16, 0.22)	0.06	0.09	0.08 (0.07, 0.09)
c. Jobs creation programs until 2024	0.54	0.87	0.74 (0.55, 0.87)	0.6	0.98	0.82 (0.61, 0.97)	0.33	0.54	0.46 (0.34, 0.54)
d. Post-suicide attempt care starting in Jan 2022	2.13	3.12	2.63 (2.15, 3.1)	2.09	3.06	2.58 (2.11, 3.04)	0.22	0.31	0.26 (0.22, 0.31)
e. Post-suicide attempt care starting in Jan 2023	1.22	1.8	1.51 (1.24, 1.79)	1.2	1.77	1.49 (1.22, 1.75)	0.12	0.18	0.15 (0.13, 0.18)
f. Post-suicide attempt care starting in Jan 2024	0.47	0.7	0.58 (0.48, 0.69)	0.46	0.68	0.57 (0.47, 0.68)	0.05	0.07	0.06 (0.05, 0.07)
g. Services capacity increase by 100% and technolo	0.7	0.93	0.81 (0.72, 0.9)	0.61	0.82	0.71 (0.64, 0.79)	1.63	2.17	1.88 (1.68, 2.08)

gy-enabled care									
h. Interventions b, c, d, g	3.62	4.92	4.22 (3.73, 4.77)	3.68	4.94	4.26 (3.75, 4.81)	2.43	3.09	2.79 (2.5, 3.04)
**Population aged 15-24 years**									
a. Awareness campaigns	-0.08	-0.01	-0.05 (-0.08, -0.02)	-0.08	-0.01	-0.05 (-0.08, -0.02)	-7.49	-5.12	-6.33 (-7.43, -5.18)
b. Education programs	0.66	0.93	0.8 (0.67, 0.92)	0.66	0.93	0.8 (0.67, 0.92)	0.42	0.59	0.51 (0.43, 0.59)
c. Jobs creation programs until 2024	0.75	1.24	1.03 (0.76, 1.23)	0.75	1.24	1.03 (0.76, 1.23)	0.44	0.74	0.61 (0.45, 0.73)
d. Post-suicide attempt care starting in Jan 2022	2.01	2.95	2.48 (2.03, 2.92)	2.01	2.95	2.48 (2.03, 2.93)	0.33	0.47	0.4 (0.34, 0.47)
e. Post-suicide attempt care starting in Jan 2023	1.17	1.72	1.44 (1.18, 1.7)	1.17	1.72	1.44 (1.18, 1.7)	0.19	0.28	0.23 (0.2, 0.27)
f. Post-suicide attempt care starting in Jan 2024	0.45	0.67	0.56 (0.46, 0.67)	0.46	0.67	0.56 (0.46, 0.67)	0.08	0.11	0.09 (0.08, 0.11)
g. Services capacity increase by 100% and technolo gy-enabled care	0.37	0.5	0.43 (0.39, 0.49)	0.37	0.5	0.43 (0.39, 0.49)	1.01	1.37	1.18 (1.06, 1.34)
h. Interventions b, c, d, g	4.04	5.38	4.72 (4.11, 5.26)	4.04	5.38	4.72 (4.11, 5.26)	2.53	3.15	2.84 (2.56, 3.11)

Supplementary Table S4. Numerical inputs and data sources. Inputs highlighted in blue were varied in the sensitivity analysis.

Input	Stratification	Value	Notes
**Population**			
Birth rate increase per year	Major cities areas	0.000005712	Estimated via constrained optimisation
	Regional and remote areas	0.000020414	Estimated via constrained optimisation
Birth rate per year initial	Major cities areas	0.013611169	Estimated via constrained optimisation
	Regional and remote areas	0.011434248	Estimated via constrained optimisation
Death rate increase per year	Major cities areas	-0.000100598	Estimated via constrained optimisation
	Regional and remote areas	-0.000087563	Estimated via constrained optimisation
Death rate per year initial	Major cities areas	0.005956262	Estimated via constrained optimisation
	Regional and remote areas	0.006691312	Estimated via constrained optimisation
Death rate ratio	Age 0-14 years	0.049953490	Estimated via constrained optimisation
	Age 15-24 years	0.049994405	Estimated via constrained optimisation
	Age 25-44 years	0.119292062	Estimated via constrained optimisation
	Age 45-64 years	0.564924026	Estimated via constrained optimisation
	Age 65 years and over	6.150733653	Estimated via constrained optimisation
Internal arrivals	Age 0-14 years, Major cities areas	236.722197299	Estimated via constrained optimisation
	Age 0-14 years, Regional and remote areas	188.098055176	Estimated via constrained optimisation
	Age 15-24 years, Major cities areas	319.900385822	Estimated via constrained optimisation
	Age 15-24 years, Regional and remote areas	177.102653874	Estimated via constrained optimisation
	Age 25-44 years, Major cities areas	594.261776421	Estimated via constrained optimisation
	Age 25-44 years, Regional and remote areas	339.697856644	Estimated via constrained optimisation
	Age 45-64 years, Major cities areas	184.427927268	Estimated via constrained optimisation
	Age 45-64 years, Regional and remote areas	179.441348232	Estimated via constrained optimisation
	Age 65 years and over, Major cities areas	61.879153446	Estimated via constrained optimisation
	Age 65 years and over, Regional and remote areas	61.325651873	Estimated via constrained optimisation
Pre-COVID overseas arrivals	Age 0-14 years, Major cities areas	290.013403688	Estimated via constrained optimisation
	Age 0-14 years, Regional and remote areas	103.020522778	Estimated via constrained optimisation
	Age 15-24 years, Major cities areas	396.209231260	Estimated via constrained optimisation
	Age 15-24 years, Regional and remote areas	221.984420299	Estimated via constrained optimisation
	Age 25-44 years, Major cities areas	862.838086731	Estimated via constrained optimisation
	Age 25-44 years, Regional and remote areas	402.522889841	Estimated via constrained optimisation
	Age 45-64 years, Major cities areas	142.038863930	Estimated via constrained optimisation
	Age 45-64 years, Regional and remote areas	110.785701628	Estimated via constrained optimisation
	Age 65 years and over, Major cities areas	30.651413734	Estimated via constrained optimisation
	Age 65 years and over, Regional and remote areas	21.549141586	Estimated via constrained optimisation
Internal departure rate	Age 0-14 years, Major cities areas	0.014912808	Estimated via constrained optimisation
	Age 0-14 years, Regional and remote areas	0.029738236	Estimated via constrained optimisation
	Age 15-24 years, Major cities areas	0.020253292	Estimated via constrained optimisation
	Age 15-24 years, Regional and remote areas	0.071824078	Estimated via constrained optimisation
	Age 25-44 years, Major cities areas	0.020077473	Estimated via constrained optimisation
	Age 25-44 years, Regional and remote areas	0.051505974	Estimated via constrained optimisation
	Age 45-64 years, Major cities areas	0.011143052	Estimated via constrained optimisation
	Age 45-64 years, Regional and remote areas	0.017048666	Estimated via constrained optimisation
	Age 65 years and over, Major cities areas	0.012961140	Estimated via constrained optimisation
	Age 65 years and over, Regional and remote areas	0.010889263	Estimated via constrained optimisation
Overseas departure rate	Age 0-14 years, Major cities areas	0.003698448	Estimated via constrained optimisation
	Age 0-14 years, Regional and remote areas	0.018402611	Estimated via constrained optimisation
	Age 15-24 years, Major cities areas	0.008809918	Estimated via constrained optimisation
	Age 15-24 years, Regional and remote areas	0.054567510	Estimated via constrained optimisation
	Age 25-44 years, Major cities areas	0.007092600	Estimated via constrained optimisation
	Age 25-44 years, Regional and remote areas	0.065991037	Estimated via constrained optimisation
	Age 45-64 years, Major cities areas	0.004423970	Estimated via constrained optimisation
	Age 45-64 years, Regional and remote areas	0.005838317	Estimated via constrained optimisation
	Age 65 years and over, Major cities areas	0.002175795	Estimated via constrained optimisation
	Age 65 years and over, Regional and remote areas	0.004545667	Estimated via constrained optimisation
Population initial	Age 0-14 years, Major cities areas	760712.8159	Derived from Australian Bureau of Statistics, Estimated Resident Population by SA2 by Sex and Age
	Age 0-14 years, Regional and remote areas	249152.6793	Derived from Australian Bureau of Statistics,

			Estimated Resident Population by SA2 by Sex and Age
	Age 15-24 years, Major cities areas	600793.9979	Derived from Australian Bureau of Statistics, Estimated Resident Population by SA2 by Sex and Age
	Age 15-24 years, Regional and remote areas	167914.9989	Derived from Australian Bureau of Statistics, Estimated Resident Population by SA2 by Sex and Age
	Age 25-44 years, Major cities areas	1289942.4090	Derived from Australian Bureau of Statistics, Estimated Resident Population by SA2 by Sex and Age
	Age 25-44 years, Regional and remote areas	309372.5856	Derived from Australian Bureau of Statistics, Estimated Resident Population by SA2 by Sex and Age
	Age 45-64 years, Major cities areas	1002741.2260	Derived from Australian Bureau of Statistics, Estimated Resident Population by SA2 by Sex and Age
	Age 45-64 years, Regional and remote areas	353081.2664	Derived from Australian Bureau of Statistics, Estimated Resident Population by SA2 by Sex and Age
	Age 65 years and over, Major cities areas	546275.3634	Derived from Australian Bureau of Statistics, Estimated Resident Population by SA2 by Sex and Age
	Age 65 years and over, Regional and remote areas	219471.6320	Derived from Australian Bureau of Statistics, Estimated Resident Population by SA2 by Sex and Age
**Psychological distress**			
Psychological distress onset base rate	Age 15-24 years, Major cities areas	0.140960248	Estimated via constrained optimisation
	Age 15-24 years, Regional and remote areas	0.141305836	Estimated via constrained optimisation
	Age 25-44 years, Major cities areas	0.092380075	Estimated via constrained optimisation
	Age 25-44 years, Regional and remote areas	0.091426952	Estimated via constrained optimisation
	Age 45-64 years, Major cities areas	0.084192614	Estimated via constrained optimisation
	Age 45-64 years, Regional and remote areas	0.079497591	Estimated via constrained optimisation
	Age 65 years and over, Major cities areas	0.067190060	Estimated via constrained optimisation
	Age 65 years and over, Regional and remote areas	0.066267902	Estimated via constrained optimisation
Psychological distress prevalence initial	Age 15-24 years, Major cities areas	0.369397785	Estimated via constrained optimisation
	Age 15-24 years, Regional and remote areas	0.362954019	Estimated via constrained optimisation
	Age 25-44 years, Major cities areas	0.283263589	Estimated via constrained optimisation
	Age 25-44 years, Regional and remote areas	0.280001814	Estimated via constrained optimisation
	Age 45-64 years, Major cities areas	0.270740469	Estimated via constrained optimisation
	Age 45-64 years, Regional and remote areas	0.268794539	Estimated via constrained optimisation
	Age 65 years and over, Major cities areas	0.199438926	Estimated via constrained optimisation
	Age 65 years and over, Regional and remote areas	0.199227962	Estimated via constrained optimisation
Effect of unemployment on psychological distress		1.732936000	Derived from Australian Bureau of Statistics (2012, Information paper. Use of the Kessler psychological distress scale in ABS health surveys, Australia, 2007-08. Cat. no. 4817.0.55.001. Australian Bureau of Statistics, Canberra)
Unemployment rate ratio non-distressed		0.696177000	Derived from Australian Bureau of Statistics (2012, Information paper. Use of the Kessler psychological distress scale in ABS health surveys, Australia, 2007-08. Cat. no. 4817.0.55.001. Australian Bureau of Statistics, Canberra)
Effect of underemployment on psychological distress		1.132448000	Derived from Dooley et al. (2000, J. Health Soc. Behav. 41, 421-436)
Underemployment rate ratio non-distressed		0.975887900	Derived from Dooley et al. (2000, J. Health Soc. Behav. 41, 421-436)
Effect of psychopathological vulnerability on distress onset		1.95	Derived from Green et al. (2019, Aust. N. Z. J. Psychiatry 53, 304-315)
Psychopathological vulnerability prevalence ratio non-distressed		0.918356100	Derived from Green et al. (2019, Aust. N. Z. J. Psychiatry 53, 304-315)
Migrant psychological distress prevalence ratio		0.798851123	Australian Bureau of Statistics (2018, National Health Survey: first results, 2017-18. Cat. no. 4364.0.55.001. Australian Bureau of Statistics, Canberra)
**Psychopathological vulnerability**			
Developing psychopathological vulnerability base rate	Age 0-14 years, Major cities areas	0.009010741	Estimated via constrained optimisation

	Age 0-14 years, Regional and remote areas	0.007094532	Estimated via constrained optimisation
Psychopathological vulnerability prevalence initial	Major cities areas	0.094140212	Estimated via constrained optimisation
	Regional and remote areas	0.093142710	Estimated via constrained optimisation
Effect of parental psychological distress on vulnerability	Age 0-14 years	1.63	Derived from Dean et al. (2018, Psychol. Med. 48, 2257-2263)
Parental psychological distress prevalence ratio not vulnerable	Age 0-14 years	0.947830700	Derived from Dean et al. (2018, Psychol. Med. 48, 2257-2263)
Proportion of population with dependent children	Age 15-24 years	0.043867662	Derived from Australian Bureau of Statistics. Family Characteristics and Transitions, Australia, 2012-13 data
	Age 25-44 years	0.542670616	Derived from Australian Bureau of Statistics. Family Characteristics and Transitions, Australia, 2012-13 data
	Age 45-64 years	0.486845213	Derived from Australian Bureau of Statistics. Family Characteristics and Transitions, Australia, 2012-13 data
	Age 65 years and over	0.178025686	Derived from Australian Bureau of Statistics. Family Characteristics and Transitions, Australia, 2012-13 data
**Education**			
Discontinuing post-secondary study base rate	Age 15-24 years	0.514843220	Estimated via constrained optimisation
	Age 25-44 years	0.355353608	Estimated via constrained optimisation
	Age 45-64 years	0.914640795	Estimated via constrained optimisation
Proportion completing first post-secondary qualification	Age 15-24 years, Major cities areas	0.392435240	Estimated via constrained optimisation
	Age 15-24 years, Regional and remote areas	0.298072867	Estimated via constrained optimisation
	Age 25-44 years, Major cities areas	0.331762633	Estimated via constrained optimisation
	Age 25-44 years, Regional and remote areas	0.133087607	Estimated via constrained optimisation
	Age 45-64 years, Major cities areas	0.060440367	Estimated via constrained optimisation
	Age 45-64 years, Regional and remote areas	0.039834123	Estimated via constrained optimisation
Starting post-secondary study base rate	Age 15-24 years, Major cities areas	0.611637308	Estimated via constrained optimisation
	Age 15-24 years, Regional and remote areas	0.417709602	Estimated via constrained optimisation
	Age 25-44 years, Major cities areas	0.098383705	Estimated via constrained optimisation
	Age 25-44 years, Regional and remote areas	0.135948492	Estimated via constrained optimisation
	Age 45-64 years, Major cities areas	0.048422416	Estimated via constrained optimisation
	Age 45-64 years, Regional and remote areas	0.078282317	Estimated via constrained optimisation
Post-secondary study proportion initial	Age 15-24 years, Major cities areas	0.355115423	Derived from Australian Bureau of Statistics, Education and Work 2019
	Age 15-24 years, Regional and remote areas	0.202388926	Derived from Australian Bureau of Statistics, Education and Work 2019
	Age 25-44 years, Major cities areas	0.111649431	Derived from Australian Bureau of Statistics, Education and Work 2019
	Age 25-44 years, Regional and remote areas	0.096690348	Derived from Australian Bureau of Statistics, Education and Work 2019
	Age 45-64 years, Major cities areas	0.034433726	Derived from Australian Bureau of Statistics, Education and Work 2019
	Age 45-64 years, Regional and remote areas	0.039115740	Derived from Australian Bureau of Statistics, Education and Work 2019
Post-secondary qualification proportion initial	Age 15-24 years, Major cities areas	0.220696711	Derived from Australian Bureau of Statistics, Education and Work 2019
	Age 15-24 years, Regional and remote areas	0.182269013	Derived from Australian Bureau of Statistics, Education and Work 2019
	Age 25-44 years, Major cities areas	0.654297384	Derived from Australian Bureau of Statistics, Education and Work 2019
	Age 25-44 years, Regional and remote areas	0.544850690	Derived from Australian Bureau of Statistics, Education and Work 2019
	Age 45-64 years, Major cities areas	0.538839012	Derived from Australian Bureau of Statistics, Education and Work 2019
	Age 45-64 years, Regional and remote areas	0.477588786	Derived from Australian Bureau of Statistics, Education and Work 2019
Death rate ratio post-secondary qualification		0.735294118	Derived from Backlund et al. (1999, Soc. Sci. Med. 49, 1373-1384)
Migrant post-secondary qualification probability ratio		1.075613702	Derived from Australian Bureau of Statistics, Migrants, Education and Work 2019
Effect of psychological distress on post-secondary education		0.833333300	Derived from Lee et al. (2009, Br. J. Psychiatry 194, 411-417)
Effect of psychological distress on discontinuation of post-secondary education		1.1	Derived from Lee et al. (2009, Br. J. Psychiatry 194, 411-417)

**Employment**			
Employed to NILF base rate	Age 15-24 years	0.230345489	Estimated via constrained optimisation
	Age 25-44 years	0.028621158	Estimated via constrained optimisation
	Age 45-64 years	0.029305930	Estimated via constrained optimisation
Employed to underemployed base rate	Age 15-24 years	0.000000000	Estimated via constrained optimisation
	Age 25-44 years	0.341915821	Estimated via constrained optimisation
	Age 45-64 years	0.365477832	Estimated via constrained optimisation
Employed to unemployed base rate	Age 15-24 years	0.169541615	Estimated via constrained optimisation
	Age 25-44 years	0.057248722	Estimated via constrained optimisation
	Age 45-64 years	0.055423857	Estimated via constrained optimisation
NILF to unemployed base rate	Age 15-24 years, Major cities areas	0.605087967	Estimated via constrained optimisation
	Age 15-24 years, Regional and remote areas	0.795900232	Estimated via constrained optimisation
	Age 25-44 years, Major cities areas	0.288692216	Estimated via constrained optimisation
	Age 25-44 years, Regional and remote areas	0.215008522	Estimated via constrained optimisation
	Age 45-64 years, Major cities areas	0.081843686	Estimated via constrained optimisation
	Age 45-64 years, Regional and remote areas	0.082773834	Estimated via constrained optimisation
Underemployed to employed base rate	Age 15-24 years	1.806575267	Estimated via constrained optimisation
	Age 25-44 years	3.463841411	Estimated via constrained optimisation
	Age 45-64 years	4.247287747	Estimated via constrained optimisation
Underemployed to NILF base rate	Age 15-24 years	0.000025982	Estimated via constrained optimisation
	Age 25-44 years	0.185647422	Estimated via constrained optimisation
	Age 45-64 years	0.032387103	Estimated via constrained optimisation
Underemployed to unemployed base rate	Age 15-24 years	0.034161371	Estimated via constrained optimisation
	Age 25-44 years	0.414877792	Estimated via constrained optimisation
	Age 45-64 years	0.194671165	Estimated via constrained optimisation
Unemployed to employed base rate	Age 15-24 years, Major cities areas	0.058392399	Estimated via constrained optimisation
	Age 15-24 years, Regional and remote areas	0.416291772	Estimated via constrained optimisation
	Age 25-44 years, Major cities areas	2.928028494	Estimated via constrained optimisation
	Age 25-44 years, Regional and remote areas	2.357894110	Estimated via constrained optimisation
	Age 45-64 years, Major cities areas	2.176342869	Estimated via constrained optimisation
	Age 45-64 years, Regional and remote areas	1.421868847	Estimated via constrained optimisation
Unemployed to NILF base rate	Age 15-24 years	3.045387470	Estimated via constrained optimisation
	Age 25-44 years	2.163056957	Estimated via constrained optimisation
	Age 45-64 years	2.128404168	Estimated via constrained optimisation
Unemployed to NILF rate coefficient unemployment	Age 15-24 years	1.663855685	Estimated via constrained optimisation
	Age 25-44 years	1.148233134	Estimated via constrained optimisation
	Age 45-64 years	1.018995294	Estimated via constrained optimisation
Unemployed to underemployed base rate	Age 15-24 years, Major cities areas	2.938718004	Estimated via constrained optimisation
	Age 15-24 years, Regional and remote areas	3.405752801	Estimated via constrained optimisation
	Age 25-44 years, Major cities areas	0.082738738	Estimated via constrained optimisation
	Age 25-44 years, Regional and remote areas	0.902704996	Estimated via constrained optimisation
	Age 45-64 years, Major cities areas	0.204494788	Estimated via constrained optimisation
	Age 45-64 years, Regional and remote areas	1.232974847	Estimated via constrained optimisation
Employed proportion initial	Age 15-24 years, Major cities areas	0.616317913	Derived from Australian Bureau of Statistics, Labour Force, Australia 2019
	Age 15-24 years, Regional and remote areas	0.618544508	Derived from Australian Bureau of Statistics, Labour Force, Australia 2019
	Age 25-44 years, Major cities areas	0.790999868	Derived from Australian Bureau of Statistics, Labour Force, Australia 2019
	Age 25-44 years, Regional and remote areas	0.775968579	Derived from Australian Bureau of Statistics, Labour Force, Australia 2019
	Age 45-64 years, Major cities areas	0.713404939	Derived from Australian Bureau of Statistics, Labour Force, Australia 2019
	Age 45-64 years, Regional and remote areas	0.695142713	Derived from Australian Bureau of Statistics, Labour Force, Australia 2019
Unemployed proportion initial	Age 15-24 years, Major cities areas	0.087697142	Derived from Australian Bureau of Statistics, Labour Force, Australia 2019
	Age 15-24 years, Regional and remote areas	0.095630003	Derived from Australian Bureau of Statistics, Labour Force, Australia 2019
	Age 25-44 years, Major cities areas	0.033621655	Derived from Australian Bureau of Statistics, Labour Force, Australia 2019
	Age 25-44 years, Regional and remote areas	0.035541734	Derived from Australian Bureau of Statistics, Labour Force, Australia 2019
	Age 45-64 years, Major cities areas	0.024077597	Derived from Australian Bureau of Statistics, Labour Force, Australia 2019
	Age 45-64 years, Regional and remote areas	0.025003664	Derived from Australian Bureau of Statistics,

			Labour Force, Australia 2019
Underemployed proportion initial	Age 15-24 years	0.162401338	Derived from Australian Bureau of Statistics, Labour Force, Australia 2019
	Age 25-44 years	0.056678129	Derived from Australian Bureau of Statistics, Labour Force, Australia 2019
	Age 45-64 years	0.058327686	Derived from Australian Bureau of Statistics, Labour Force, Australia 2019
Migrant underemployment probability ratio		0.862288655	Derived from Wilkins (2006, Aust. J. Labour Econ. 9, 371-393)
Migrant unemployment probability ratio		0.994422716	Derived from Australian Bureau of Statistics, Labour Force Detailed, Australia 2019
Migrant employment probability ratio		0.956702207	Derived from Australian Bureau of Statistics, Labour Force Detailed, Australia 2019
Effect of psychological distress on participation		0.839659600	Derived from Frijters et al. (2014, Health Econ. 23, 1058−1071)
Effect of psychological distress on employment		0.839659600	Derived from Frijters et al. (2014, Health Econ. 23, 1058−1071)
Effect of post-secondary education on participation		1.351342518	Derived from Australian Bureau of Statistics (2020, Education and work, Australia, May 2020. Cat. no. 6227.0. Australian Bureau of Statistics, Canberra)
Effect of post-secondary education on employment		1.046560897	Derived from Australian Bureau of Statistics (2020, Education and work, Australia, May 2020. Cat. no. 6227.0. Australian Bureau of Statistics, Canberra)
Death rate ratio unemployed		1.22	Derived from Sorlie and Rogot (1990, Am. J. Epidemiol. 132, 983−992)
Post-secondary qualification probability ratio NILF		0.679195716	Derived from Australian Bureau of Statistics (2020, Education and work, Australia, May 2020. Cat. no. 6227.0. Australian Bureau of Statistics, Canberra)
Post-secondary qualification probability ratio underemployed		0.862828720	Derived from Wilkins (2004, The extent and consequences of underemployment in Australia. Melbourne Institute working paper no. 16/04. The University of Melbourne, Melbourne) and Wilkins (2006, Aust. J. Labour Econ. 9, 371-393)
Post-secondary study employed proportion ratio	Age 15-24 years, Major cities areas	0.519269670	Estimated via constrained optimisation
	Age 15-24 years, Regional and remote areas	0.766553475	Estimated via constrained optimisation
Unemployed to NILF rate coefficient unemployment	Age 15-24 years	1.663855685	Estimated via constrained optimisation
	Age 25-44 years	1.148233134	Estimated via constrained optimisation
	Age 45-64 years	1.018995294	Estimated via constrained optimisation
Effect of education on underemployed to employed rate		1.407043821	Derived from Wilkins (2004, The extent and consequences of underemployment in Australia. Melbourne Institute working paper no. 16/04. The University of Melbourne, Melbourne) and Wilkins (2006, Aust. J. Labour Econ. 9, 371-393)
**Suicidal behaviour**			
Self-harm hospitalisation rate non-distressed	Age 0-14 years, Major cities areas	0.000069607	Estimated via constrained optimisation
	Age 0-14 years, Regional and remote areas	0.000168588	Estimated via constrained optimisation
	Age 15-24 years, Major cities areas	0.000372407	Estimated via constrained optimisation
	Age 15-24 years, Regional and remote areas	0.000675128	Estimated via constrained optimisation
	Age 25-44 years, Major cities areas	0.000226430	Estimated via constrained optimisation
	Age 25-44 years, Regional and remote areas	0.000491479	Estimated via constrained optimisation
	Age 45-64 years, Major cities areas	0.000194368	Estimated via constrained optimisation
	Age 45-64 years, Regional and remote areas	0.000288011	Estimated via constrained optimisation
	Age 65 years and over, Major cities areas	0.000082107	Estimated via constrained optimisation
	Age 65 years and over, Regional and remote areas	0.000087650	Estimated via constrained optimisation
Suicide attempt lethality initial	Age 0-14 years	0.021220755	Estimated via constrained optimisation
	Age 15-24 years	0.052111011	Estimated via constrained optimisation
	Age 25-44 years	0.123474937	Estimated via constrained optimisation
	Age 45-64 years	0.168378275	Estimated via constrained optimisation
	Age 65 years and over	0.358069053	Estimated via constrained optimisation
Suicide attempt lethality multiplier increase per year		-0.000428121	Estimated via constrained optimisation
**Services**			

CMHC services capacity increase per year	Major cities areas	407.303967750	Estimated via constrained optimisation
	Regional and remote areas	24.449580592	Estimated via constrained optimisation
CMHC services capacity initial	Major cities areas	20170.471290400	Estimated via constrained optimisation
	Regional and remote areas	8362.132220490	Estimated via constrained optimisation
GP services capacity increase per year	Major cities areas	979.474090885	Estimated via constrained optimisation
	Regional and remote areas	324.340639919	Estimated via constrained optimisation
GP services capacity initial	Major cities areas	7818.335290520	Estimated via constrained optimisation
	Regional and remote areas	2961.383353070	Estimated via constrained optimisation
Non-specialised hospital capacity increase per year	Major cities areas	9.919428365	Estimated via constrained optimisation
	Regional and remote areas	1.252002347	Estimated via constrained optimisation
Non-specialised hospital capacity initial	Major cities areas	138.912829802	Estimated via constrained optimisation
	Regional and remote areas	89.802449675	Estimated via constrained optimisation
Private hospital capacity increase per year	Major cities areas	10.007322791	Estimated via constrained optimisation
	Regional and remote areas	3.453473891	Estimated via constrained optimisation
Private hospital capacity initial	Major cities areas	141.961220253	Estimated via constrained optimisation
	Regional and remote areas	59.115084832	Estimated via constrained optimisation
Private outpatient services capacity increase per year	Major cities areas	15.273014175	Estimated via constrained optimisation
	Regional and remote areas	10.146706910	Estimated via constrained optimisation
Private outpatient services capacity initial	Major cities areas	1434.729354560	Estimated via constrained optimisation
	Regional and remote areas	47.552058868	Estimated via constrained optimisation
Psychiatric hospital capacity increase per year	Major cities areas	12.718616160	Estimated via constrained optimisation
	Regional and remote areas	4.928750998	Estimated via constrained optimisation
Psychiatric hospital capacity initial	Major cities areas	229.726659250	Estimated via constrained optimisation
	Regional and remote areas	83.605232465	Estimated via constrained optimisation
Psychiatrist and allied services capacity increase per year	Major cities areas	1468.119047610	Estimated via constrained optimisation
	Regional and remote areas	492.542641723	Estimated via constrained optimisation
Psychiatrist and allied services capacity initial	Major cities areas	25683.101715000	Estimated via constrained optimisation
	Regional and remote areas	6515.546704950	Estimated via constrained optimisation
Mean treatment duration non-specialised hospital care		0.883845357	Derived from national data on mental health-related hospitalisations published by the Australian Institute of Health and Welfare (available at: https://www.aihw.gov.au/reports-data/health-welfare-services/mental-health-services/data)
Effect of psychological distress on help seeking	High distress	0.950333300	Derived from Australian Bureau of Statistics (2012, Information paper. Use of the Kessler psychological distress scale in ABS health surveys, Australia, 2007-08. Cat. no. 4817.0.55.001. Australian Bureau of Statistics, Canberra)
	Low distress	1	Reference category
Mean treatment duration online services		6	Derived from Christensen et al. (2004, Br. Med. J. 328, 265)
Referral rate online services		0.046749000	Derived from national data on mental health-related general practitioner services published by the Australian Institute of Health and Welfare (available at: https://www.aihw.gov.au/reports-data/health-welfare-services/mental-health-services/data)
Mean treatment duration private hospital		2.526374701	Derived from national data on mental health-related hospitalisations published by the Australian Institute of Health and Welfare (available at: https://www.aihw.gov.au/reports-data/health-welfare-services/mental-health-services/data)
Effect of disengagement on recovery		0.454296830	Derived from Australian Bureau of Statistics (2012, Information paper. Use of the Kessler psychological distress scale in ABS health surveys, Australia, 2007-08. Cat. no. 4817.0.55.001. Australian Bureau of Statistics, Canberra)
Effect of disengagement on psychological distress		2.201204000	Derived from Australian Bureau of Statistics (2012, Information paper. Use of the Kessler psychological distress scale in ABS health

			surveys, Australia, 2007-08. Cat. no. 4817.0.55.001. Australian Bureau of Statistics, Canberra)
Disengaged to perceived need for services rate		5.181821348	Estimated via constrained optimisation
Disengagement rate waiting		0.2620284	Derived from Tyrer et al. (1995, Lancet 345, 756−759)
Disengagement rate hospital care		0.051642558	Derived from state-level consumer survey data for 2016-17 published by the Australian Institute of Health and Welfare (available at: https://www.aihw.gov.au/reports-data/health-welfare-services/mental-health-services/data)
Disengagement rate non-hospital care		0.03909747	Derived from state-level consumer survey data for 2016-17 published by the Australian Institute of Health and Welfare (available at: https://www.aihw.gov.au/reports-data/health-welfare-services/mental-health-services/data)
Natural recovery rate ratio low distress		3.225351000	Derived from Kessler et al. (1997, J. Affect. Disord. 45, 19-30)
Effect of psychological distress on ED presentation rate	High distress	7.420126000	Derived from Australian Bureau of Statistics (2012, Information paper. Use of the Kessler psychological distress scale in ABS health surveys, Australia, 2007-08. Cat. no. 4817.0.55.001. Australian Bureau of Statistics, Canberra)
	Low distress	1	Reference category
Post-discharge non-CMHC services referral proportion GP		0.5	Assumes half of patients not referred to CMHC services after discharge from hospital care are referred to a general practitioner. The remaining patients (i.e., those not referred to CMHC services or a GP) are referred to a psychiatrist or allied mental health professional.
Effect of psychological distress on non-specialised hospitalisation rate	High distress	7.420126000	Derived from Australian Bureau of Statistics (2012, Information paper. Use of the Kessler psychological distress scale in ABS health surveys, Australia, 2007-08. Cat. no. 4817.0.55.001. Australian Bureau of Statistics, Canberra)
	Low distress	1	Reference category
Recovery base rate CMHC services		0.025450458	Per-service recovery rate derived from data on patient outcomes and numbers of services per patient per year published online by the Australian Institute of Health and Welfare (https://www.aihw.gov.au/reports-data/health-welfare-services/mental-health-services/data)
Recovery rate online services	High distress	0.185074640	Derived from Christensen et al. (2004, Br. Med. J. 328, 265) and Cuijpers et al. (2009, Br. J. Gen. Pract., doi: 10.3399/bjgp09X395139)
	Low distress	0.400000000	Derived from Christensen et al. (2004, Br. Med. J. 328, 265)
Psychological treatment rate GP services		0.483438750	Derived from national data on mental health-related general practitioner services published by the Australian Institute of Health and Welfare (available at: https://www.aihw.gov.au/reports-data/health-welfare-services/mental-health-services/data)
Recovery rate ratio GP services	High distress	0.462686600	Derived from Cuijpers et al. (2009, Br. J. Gen. Pract., doi: 10.3399/bjgp09X395139)
	Low distress	1	Reference category
CMHC services referral rate increase per year		0.085217552	Estimated via constrained optimisation
CMHC services referral rate initial		0.527696518	Estimated via constrained optimisation
Hospital admission rate increase per year		0.021791898	Estimated via constrained optimisation
Hospital admission rate initial		0.331107719	Estimated via constrained optimisation
Referral rate psychiatrist and allied services increase per year		0.005955229	Estimated via constrained optimisation
Referral rate psychiatrist or allied services initial		0.071123168	Estimated via constrained optimisation
Seeking help GP services base rate age array	Age 0-14 years	0.366495967	Estimated via constrained optimisation
	Age 15-24 years	1.115622289	Estimated via constrained optimisation
	Age 25-44 years	2.293651367	Estimated via constrained optimisation

	Age 45-64 years	2.204450906	Estimated via constrained optimisation
	Age 65 years and over	0.471827799	Estimated via constrained optimisation
Seeking help GP services rate increase per year		0.040000385	Estimated via constrained optimisation
Seeking help GP services rate ratio regional	Major cities areas	1.000000000	Estimated via constrained optimisation
	Regional and remote areas	1.508282829	Estimated via constrained optimisation
Waiting for GP mental health services total initial		0.188491369	Estimated via constrained optimisation
Disengaged to perceived need for services rate		5.181821348	Estimated via constrained optimisation
Perceived need for services low distress proportion initial		0.362636873	Estimated via constrained optimisation
Perceived need for services total initial		748084.222015000	Estimated via constrained optimisation
Perceiving need for services base rate	High psychological distress	0.187162834	Estimated via constrained optimisation
	Low psychological distress	0.033458475	Estimated via constrained optimisation
Perceiving need for services rate increase per year		0.007467334	Estimated via constrained optimisation
Additional psychiatrist and allied services rate ratio regional	Major cities areas	1	
	Regional and remote areas	1.088809900	Estimated via constrained optimisation
Additional psychiatrist or allied services rate age array	Age 0-14 years	1.640533470	Estimated via constrained optimisation
	Age 15-24 years	3.256171142	Estimated via constrained optimisation
	Age 25-44 years	6.921246425	Estimated via constrained optimisation
	Age 45-64 years	7.400782920	Estimated via constrained optimisation
	Age 65 years and over	1.371005333	Estimated via constrained optimisation
Waiting for psychiatrist or allied services total initial		0	Estimated via constrained optimisation
Additional CMHC service contacts rate age array	Age 0-14 years	0.713176065	Estimated via constrained optimisation
	Age 15-24 years	2.546250034	Estimated via constrained optimisation
	Age 25-44 years	4.661268738	Estimated via constrained optimisation
	Age 45-64 years	4.104389999	Estimated via constrained optimisation
	Age 65 years and over	1.365034230	Estimated via constrained optimisation
CMHC services referral rate ED		1	Estimated via constrained optimisation
ED presentation base rate	Major cities areas	0.001085139	Estimated via constrained optimisation
	Regional and remote areas	0.002038685	Estimated via constrained optimisation
ED presentation rate ratio age	Age 0-14 years	0.609063474	Estimated via constrained optimisation
	Age 15-24 years	2.077700580	Estimated via constrained optimisation
	Age 25-44 years	2.211833522	Estimated via constrained optimisation
	Age 45-64 years	1.435328898	Estimated via constrained optimisation
ED presentation rate ratio perceived need for services		2.468338361	Estimated via constrained optimisation
Private hospital referral rate age array	Age 0-14 years	0.001210065	Estimated via constrained optimisation
	Age 15-24 years	0.022341652	Estimated via constrained optimisation
	Age 25-44 years	0.043353931	Estimated via constrained optimisation
	Age 45-64 years	0.035639274	Estimated via constrained optimisation
	Age 65 years and over	0.013452506	Estimated via constrained optimisation
Private hospital referral rate ratio regional	Major cities areas	1	
	Regional and remote areas	1.532587690	Estimated via constrained optimisation
Additional admission rate non-specialised hospital care age array	Age 0-14 years	0.001509506	Estimated via constrained optimisation
	Age 15-24 years	0.001911865	Estimated via constrained optimisation
	Age 25-44 years	0.005015641	Estimated via constrained optimisation
	Age 45-64 years	0.004776943	Estimated via constrained optimisation
	Age 65 years and over	0.006607063	Estimated via constrained optimisation
Additional non-specialised hospitalisations rate ratio regional	Major cities areas	1	
	Regional and remote areas	1.198628142	Estimated via constrained optimisation
Psychiatric hospital admission proportion	Age 0-14 years	0.095543582	Estimated via constrained optimisation
	Age 15-24 years	0.622199663	Estimated via constrained optimisation
	Age 25-44 years	0.694479604	Estimated via constrained optimisation
	Age 45-64 years	0.656433086	Estimated via constrained optimisation
	Age 65 years and over	0.353545688	Estimated via constrained optimisation
Referred to psychiatric hospital rate	Age 0-14 years	0.000141380	Estimated via constrained optimisation
	Age 15-24 years	0.004094285	Estimated via constrained optimisation

	Age 25-44 years	0.004779456	Estimated via constrained optimisation
	Age 45-64 years	0.004889960	Estimated via constrained optimisation
	Age 65 years and over	0.008968471	Estimated via constrained optimisation
Private outpatient services referral rate age array	Age 0-14 years	0.000307916	Estimated via constrained optimisation
	Age 15-24 years	0.103043506	Estimated via constrained optimisation
	Age 25-44 years	0.259690333	Estimated via constrained optimisation
	Age 45-64 years	0.367715161	Estimated via constrained optimisation
	Age 65 years and over	0.087254594	Estimated via constrained optimisation
Private outpatient services referral rate ratio regional	Major cities areas	1	
	Regional and remote areas	0.087856730	Estimated via constrained optimisation
**Impacts of COVID**			
Maximum decrease in overseas arrivals due to COVID		0.213556845	Estimated via constrained optimisation
Overseas migration effect duration		10.207945386	Estimated via constrained optimisation
Migration effect starting year		2020.167	Equivalent to March 2020
Employed to underemployed rate multiplier ratio	Age 15-24 years	0.484686584	Estimated via constrained optimisation
	Age 25-44 years	1.418987806	Estimated via constrained optimisation
	Age 45-64 years	1.394526689	Estimated via constrained optimisation
Unemployment effect decay rate		0.090117940	Estimated via constrained optimisation
Unemployment increase effect	Age 15-24 years, Major cities areas	2.961413233	Estimated via constrained optimisation
	Age 15-24 years, Regional and remote areas	1.988809800	Estimated via constrained optimisation
	Age 25-44 years, Major cities areas	2.905926242	Estimated via constrained optimisation
	Age 25-44 years, Regional and remote areas	2.139615477	Estimated via constrained optimisation
	Age 45-64 years, Major cities areas	3.054289606	Estimated via constrained optimisation
	Age 45-64 years, Regional and remote areas	1.616352678	Estimated via constrained optimisation
Unemployment increase starting year		2020.167	Equivalent to March 2020
Years to increase unemployment		0.002000000	Estimated via constrained optimisation
Maximum decrease in services provision due to COVID		0.739989534	Estimated via constrained optimisation
Services effect starting year		2020.167	Equivalent to March 2020
Services effect duration		2.509911587	Estimated via constrained optimisation
Effect of doubling psychological distress prevalence on recovery		400.065660650	Estimated via constrained optimisation
Sense of Community Index decrease	Age 15-24 years	0.801450844	Estimated via constrained optimisation
	Age 25-44 years	0.681282076	Estimated via constrained optimisation
	Age 45-64 years	0.457028364	Estimated via constrained optimisation
	Age 65 years and over	0	Estimated via constrained optimisation
Social connectedness decay rate		0.745789758	Estimated via constrained optimisation
Social dislocation duration		0.324297202	Estimated via constrained optimisation
Years to reach minimum Sense of Community Index		0.060984348	Estimated via constrained optimisation
Social dislocation starting year		2020.167	Equivalent to March 2020
Sense of Community Index initial		9.149557522	Derived from Handley et al. (2012, Soc. Psychiatry Psychiatr. Epidemiol. 47, 1281−1290)
Effect of Sense of Community Index increase on distress		0.64	Derived from Handley et al. (2012, Soc. Psychiatry Psychiatr. Epidemiol. 47, 1281−1290)
Effect of doubling unemployment on psychological distress		1.349859	Derived from Dooley et al., 1988. J. Soc. Issues 44, 107-123
Effect of doubling unemployment on unemployment effect		1.000000000	Derived from Dooley et al., 1988. J. Soc. Issues 44, 107-123
Post-secondary study proportion employed		0.678309365	Derived from Australian Bureau of Statistics (2020, Education and work, Australia, May 2020. Cat. no. 6227.0. Australian Bureau of Statistics, Canberra)
Pre-intervention proportion discontinuing study unemployed		0.5	Assumes that half of post-secondary students becoming unemployed due to the COVID-19 pandemic will discontinue study
Pre-COVID recovery base rate	Age 15-24 years	0.06833333	Derived from Jokela et al. (2011, J. Affect. Disord. 130, 454-461)
	Age 25-44 years	0.06833333	Derived from Jokela et al. (2011, J. Affect. Disord. 130, 454-461)
	Age 45-64 years	0.06833333	Derived from Jokela et al. (2011, J. Affect. Disord. 130, 454-461)
	Age 65 years and over	0.06833333	Derived from Jokela et al. (2011, J. Affect. Disord. 130, 454-461)

Pre-COVID self-harm hospitalisation rate ratio psychological distress		10.00004	Derived from Chamberlain et al. (2009, Crisis 30, 39−42)
**Intervention: Employment programs**			
Employment programs starting year		2020.333000000	Equivalent to May 2020
Years to implement employment programs		0.166666667	Equivalent to two months
Employment programs duration		0.916666667	Equivalent to eleven months. Reflects employment programs ending in April 2021
Effect of employment programs on employed to unemployed rate		0.56	Derived from Business Indicators, Business Impacts of COVID-19 (ABS survey, April 2020)
Effect of employment programs on employment initiation rate		1	Equivalent to no effect of employment programs on employment initiation
**Intervention: Better Access**			
Services per patient increase starting year		2020.75	Equivalent to October 2020 as additional COVID-19 MBC mental health support commenced then
Years to implement services per patient increase		0.166666667	Equivalent to two months
Services per patient increase duration		1.75	Additional COVID-19 MBS mental health support to end on 30 Jun 2022
Proportion of specialised services provided by psychiatrists	Major cities areas	0.376508398	Derived from data on Medicare-subsidised mental health services published by the Australian Institute of Health and Welfare (available at: https://www.aihw.gov.au/reports-data/health-welfare-services/mental-health-services/data)
	Regional and remote areas	0.279133644	Derived from data on Medicare-subsidised mental health services published by the Australian Institute of Health and Welfare (available at: https://www.aihw.gov.au/reports-data/health-welfare-services/mental-health-services/data)
Pre-intervention services per patient	Major cities areas	5.13103948	Derived from data on Medicare-subsidised mental health services published by the Australian Institute of Health and Welfare (available at: https://www.aihw.gov.au/reports-data/health-welfare-services/mental-health-services/data)
	Regional and remote areas	4.073157206	Derived from data on Medicare-subsidised mental health services published by the Australian Institute of Health and Welfare (available at: https://www.aihw.gov.au/reports-data/health-welfare-services/mental-health-services/data)
Additional psychiatrist and allied services per patient		4	Patients will attend an additional 4 consultations per year when the cap on the number of consultations per patient is increased.
Better Access services per week		1	Patients attend 1 consultation per week
**Intervention: Awareness campaigns**			
Mental health education programs starting year		2022	Equivalent to January 2022
Years to implement mental health education programs		0.1666667	Equivalent to two months
Mental health education programs duration		5	Equivalent to five years
Effect of mental health education on engagement		1.585327	Derived from Jorm et al. (2003, Psychol. Med. 33, 1071-1079).
Effect of mental health education initial		1	
Effect of mental health education decay rate		1	
**Intervention: Education programs**			
Education programs starting year		2022	Equivalent to January 2022
Years to implement education programs		0.1666667	Equivalent to two months
Education programs duration		5	Equivalent to five years
Effect of education programs on discontinuation rate		0.1	Reduce the proportion of students discontinuing study after becoming unemployed by 90%.
Effect of education programs on post-secondary study commencement rate 15-24		2	Equivalent to a increase of per capita enrolment rate by 100%
**Intervention: Jobs creation programs**			
Jobs creation programs starting year		2022	Equivalent to January 2022

Years to implement Jobs creation programs		0.1666667	Equivalent to two months
Jobs creation programs duration		2	Equivalent to two years
Effect on employment initiation		2	Increase the rate of employment initiation by 100%
**Intervention: Services capacity growth**			
GP services capacity increase starting year		2022	Equivalent to January 2022
Psychiatrist and allied services capacity increase starting year		2022	Equivalent to January 2022
CMHC services capacity increase starting year		2022	Equivalent to January 2022
Post-intervention GP services capacity increase per year multiplier		2	Increase capacity growth by 100%
Post-intervention psychiatrist and allied services capacity increase per year multiplier		2	Increase capacity growth by 100%
Post-intervention CMHC services capacity increase per year multiplier		2	Increase capacity growth by 100%
**Intervention: Technology-enabled care**			
Technology-enabled care starting year		2022	Equivalent to January 2022
Years to implement technology-enabled care		2	Equivalent to two years
Technology-enabled care duration		999	Equivalent to 999 years
Maximum technology-enabled care rate per service		0.7	The default value (0.7) assumes that technology-enabled, measurement-based care will be provided in 70% of mental health services completed when fully implemented.
Effect of technology-enabled care on referrals to specialised care	Low psychological distress	1	
	High psychological distress	1.265913	Derived from Badamgarav et al. (2003, Am. J. Psychiatry 160, 2080-2090).
Effect of technology-enabled care on recovery rate		1.177321	Derived from Woltmann et al. (2012, Am. J. Psychiatry 169, 790-804)
Effect of technology-enabled care on disengagement		0.5204988	Derived from Badamgarav et al. (2003, Am. J. Psychiatry 160, 2080-2090).
Pre-intervention recovery rate mental health treatment	Low psychological distress	0.087421601	Per-service recovery rates derived from treatment effectiveness estimates reported in Thase et al. (1997, Arch. Gen. Psychiatry 54, 1009−1015) and data on numbers of services per patient per year published online by the Australian Institute of Health and Welfare (https://www.aihw.gov.au/reports/primary-health-care/medicare-subsidised-gp-allied-health-and-specialis/data).
	High psychological distress	0.076531002	Per-service recovery rates derived from treatment effectiveness estimates reported in Thase et al. (1997, Arch. Gen. Psychiatry 54, 1009−1015) and data on numbers of services per patient per year published online by the Australian Institute of Health and Welfare (https://www.aihw.gov.au/reports/primary-health-care/medicare-subsidised-gp-allied-health-and-specialis/data).
**Intervention: Post-suicide attempt care**			
Post-attempt care starting year		2022	Equivalent to January 2022
Years to implement post-attempt care		2	Equivalent to two years
Post-attempt care program duration		999	Equivalent to 999 years
Post-attempt care effect estimate		0.3975155	The default estimate is derived from Hvid et al. (2011, Nord. J. Psychiatry 65, 292-298).
Maximum post-attempt care rate		0.7	Assumes that post-attempt care will be provided to 70% of patients hospitalised for a suicide attempt when post-attempt care programs are fully implemented.
Post-attempt care effect duration		52.14285714	Equivalent to one calendar year
Repeat attempt rate per year		0.179000000	Derived from Carroll et al. (2014, PLoS ONE 9, e89944).

REFERENCES

Australian Bureau of Statistics. Business Indicators, Business Impacts of COVID-19. April 2020. Available online: https://www.abs.gov.au/statistics/economy/business-indicators/business-conditions-and-sentiments/apr-2020 (accessed 30 May 2020).

Badamgarav E, Weingarten SR, Henning JM, Knight K, Hasselblad V, Gano A, Jr., Ofman JJ. Effectiveness of disease management programs in depression: a systematic review. Am J Psychiatry. 2003;160(12):2080-2090.

Carroll R, Metcalfe C, Gunnell D. Hospital presenting self-harm and risk of fatal and non-fatal repetition: systematic review and meta-analysis. PLoS One. 2014;9(2):e89944.

Hvid M, Vangborg K, Sorensen HJ, Nielsen IK, Stenborg JM, Wang AG. Preventing repetition of attempted suicide--II. The Amager project, a randomized controlled trial. Nord J Psychiatry. 2011;65(5):292-298.

Jorm AF, Griffiths KM, Christensen H, Korten AE, Parslow RA, Rodgers B. Providing information about the effectiveness of treatment options to depressed people in the community: a randomized controlled trial of effects on mental health literacy, help-seeking and symptoms. Psychol Med. 2003;33(6):1071-1079.

Woltmann E, Grogan-Kaylor A, Perron B, Georges H, Kilbourne AM, Bauer MS. Comparative effectiveness of collaborative chronic care models for mental health conditions across primary, specialty, and behavioral health care settings: systematic review and meta-analysis. Am J Psychiatry. 2012;169(8):790-804.
